# Myeloid-Derived Suppressor-like Cells as a Prognostic Marker in Critically Ill Patients: Insights from Experimental Endotoxemia and Intensive Care Patients

**DOI:** 10.3390/cells13040314

**Published:** 2024-02-08

**Authors:** Irene T. Schrijver, Jacobus Herderschee, Charlotte Théroude, Antonios Kritikos, Guus Leijte, Didier Le Roy, Maelick Brochut, Jean-Daniel Chiche, Matthieu Perreau, Giuseppe Pantaleo, Benoit Guery, Matthijs Kox, Peter Pickkers, Thierry Calandra, Thierry Roger

**Affiliations:** 1 Infectious Diseases Service, Department of Medicine, Lausanne University Hospital and University of Lausanne, 1011 Lausanne, Switzerlandmaelick.brochut@chuv.ch (M.B.);; 2 Radboud Center for Infectious Diseases, Radboud University Medical Center, 6525 GA Nijmegen, The Netherlands; 3 Department of Intensive Care Medicine, Radboud University Medical Center, 6525 EP Nijmegen, The Netherlands; 4 Service of Adult Intensive Care Medicine, Lausanne University Hospital and University of Lausanne, 1011 Lausanne, Switzerland; 5 Service of Immunology and Allergy, Department of Medicine, Lausanne University Hospital and University of Lausanne, 1010 Lausanne, Switzerland

**Keywords:** myeloid-derived suppressor cell, biomarker, endotoxemia, critically ill patient, intensive care, sepsis, hospital acquired pneumoniae

## Abstract

Patients admitted to the intensive care unit (ICU) often experience endotoxemia, nosocomial infections and sepsis. Polymorphonuclear and monocytic myeloid-derived suppressor cells (PMN-MDSCs and M-MDSCs) can have an important impact on the development of infectious diseases, but little is known about their potential predictive value in critically ill patients. Here, we used unsupervised flow cytometry analyses to quantify MDSC-like cells in healthy subjects challenged with endotoxin and in critically ill patients admitted to intensive care units and at risk of developing infections. Cells phenotypically similar to PMN-MDSCs and M-MDSCs increased after endotoxin challenge. Similar cells were elevated in patients at ICU admission and normalized at ICU discharge. A subpopulation of M-MDSC-like cells expressing intermediate levels of CD15 (CD15^int^ M-MDSCs) was associated with overall mortality (*p* = 0.02). Interestingly, the high abundance of PMN-MDSCs and CD15^int^ M-MDSCs was a good predictor of mortality (*p* = 0.0046 and 0.014), with area under the ROC curve for mortality of 0.70 (95% CI = 0.4–1.0) and 0.86 (0.62–1.0), respectively. Overall, our observations support the idea that MDSCs represent biomarkers for sepsis and that flow cytometry monitoring of MDSCs may be used to risk-stratify ICU patients for targeted therapy.

## 1. Introduction

Sepsis is a heterogeneous syndrome defined as a life-threatening organ dysfunction caused by a dysregulated host response to infection [1]. Despite major improvements in patient care, the incidence of sepsis is rising. The Global Burden of Disease Study estimated that sepsis affects around 49 million people and is accountable for 11 million deaths per year, representing close to 20% of all deaths worldwide. Moreover, almost half of patients surviving sepsis are re-hospitalized within a year. Hence, sepsis is a leading cause of critical illness and mortality worldwide [2]. Sepsis is accompanied by immune alterations affecting the innate and adaptive arms of the immune system. At the outset, septic patients exhibit signs of exacerbated proinflammatory responses associated with organ failure, followed by counter-regulatory immune modulating mechanisms that result in immunoparalysis and the development of secondary infections. Unfortunately, despite numerous clinical trials, this knowledge has yet to be translated into clinical application [3,4,5,6,7,8,9,10].

Myeloid-derived suppressor cells (MDSCs) are immature myeloid cells arising from the bone marrow and displaying immunosuppressive functions [11,12]. MDSCs are divided into two major subsets: polymorphonuclear MDSCs (PMN-MDSCs) defined as low-density granulocytes or as CD11b^+^ CD14^−^ CD15^+^ CD16^low/intermediate^ CD33^+^ CD66b^+^ cells, and monocytic MDSCs (M-MDSCs) defined as CD11b^+^ CD14^+^ CD15^−^ CD33^+^ HLA-DR^low/negative^ cells [11,13]. In addition, a population of early-stage MDSCs (eMDSCs) that do not express lineage markers has been reported [13,14,15]. MDSCs are barely detectable in the peripheral blood of healthy subjects, but their abundance increases under myelopoiesis-stimulating conditions such as inflammation and cancer. The great majority of our knowledge of MDSCs has been obtained in the field of oncology. MDSCs restrain innate and adaptive immune responses through the expression of arginase 1, programmed cell death ligand-1, reactive oxygen and nitrogen species (ROS and RNS), interleukin (IL)-10, transforming growth factor-β or lactate and the activation of T regulatory cells [12,16,17]. MDSCs are enriched in the tumor environment and can become one of the main leukocyte subtypes in the peripheral blood of cancer patients. Targeting MDSCs is thus considered for several diseases, and clinical trials against MDSCs have shown promising results in cancer [17,18]. The role of MDSCs in infection and sepsis is incompletely understood. In general, it is assumed that the immunosuppressive functions of MDSCs are detrimental to host defenses. Yet, MDSCs are phagocytic cells producing high amounts of bactericidal molecules such as ROS and RNS, thus actively participating in host defenses. In addition, through their regulatory functions, MDSCs may counterbalance the detrimental inflammatory response occurring during sepsis [6,16,17,19,20,21,22,23,24,25].

In-hospital mortality of intensive care unit (ICU) patients ranges from 7% to 40% and is frequently associated with nosocomial infections and sepsis. Moreover, endotoxemia is common in ICU patients [1,26]. Rapidly measurable prognostic biomarkers would be invaluable to risk-stratify critically ill patients to select and/or adapt treatment options [8,27,28,29,30]. In the present study, we aimed to assess whether MDSCs represent biomarkers in sepsis using unsupervised flow cytometry to quantify MDSC-like cells. We first applied our approach to the model of experimental human endotoxemia, a well-controlled model of acute systemic inflammation in healthy volunteers, in order to find clues about the impact of an acute inflammatory response on the expression of MDSC-like cells. Second, we analyzed the expression of MDSC-like cells in non-infectious critically ill patients admitted to the ICU with a high likelihood of developing infection.

## 2. Materials and Methods

### 2.1. Ethics, Subjects, and Study Design

The endotoxemia study was conducted at the Radboud University Nijmegen Medical Centre, Nijmegen, the Netherlands. Eight healthy male volunteers were challenged with 2 ng/kg of *Escherichia coli* O:113 lipopolysaccharide (LPS, lot #94332B1, National Institutes of Health, Bethesda, MD, USA) following a standard protocol [31]. Blood was collected in EDTA tubes before endotoxin administration (baseline) and 1, 2, 3, 4, 6, 8, 24,168 and 336 h after endotoxin administration. The PIPOVAP (Profile, Interaction, and PrOgnosis in Ventilator-Associated Pneumonia) study was a prospective, observational study conducted at the Lausanne Hospital University, Lausanne, Switzerland. Thirty-three non-infected, intubated adult patients admitted to the ICU with an anticipated length of mechanical ventilation greater than 48 h were included. Exclusion criteria were treatment with immunosuppressive agents or treatment with antibiotics. Blood was collected in EDTA tubes and serum tubes within 24 h of admission to the ICU and at discharge.

### 2.2. Flow Cytometry

**Figure 1:**
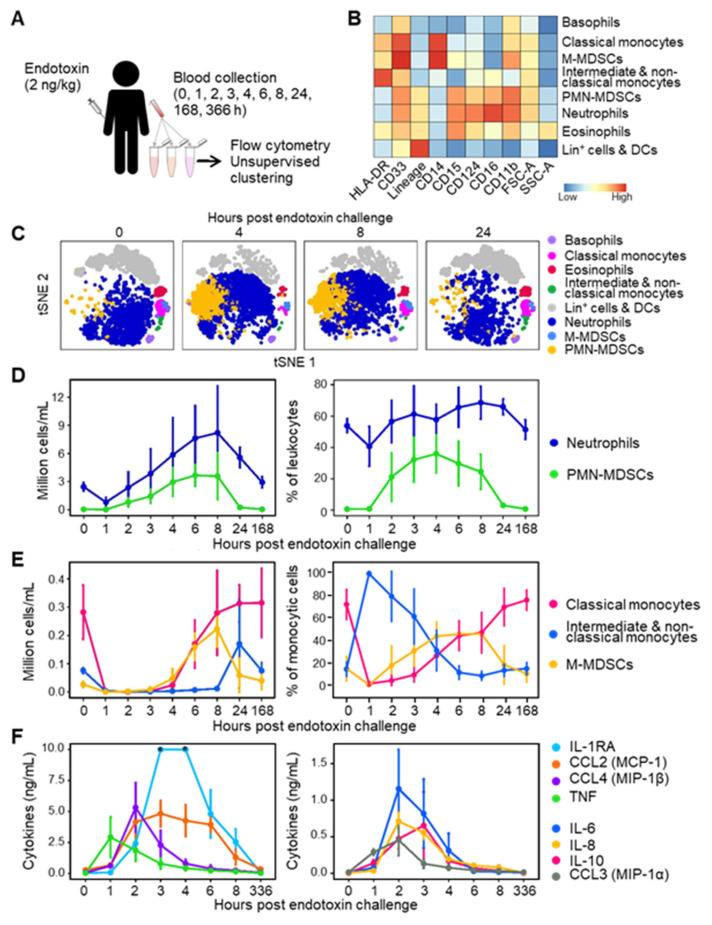
MDSC-like cells in the blood of healthy subjects challenged with endotoxin. (**A**) Study design. Eight healthy subjects were challenged with 2 ng/kg endotoxin. Blood was collected just before and 1–336 h after endotoxin infusion in DURAClone tubes and analyzed via flow cytometry. (**B**) Relative expression levels of cell surface markers and forward and side scatter areas (FSC-A, SSC-A) of leukocyte populations. (**C**) t-SNE plots of leukocyte populations over time. (**D**) Absolute counts and percentage in leukocytes of PMN-MDSCs and neutrophils. (**E**) Absolute counts and percentage in monocytic cells of M-MDSCs, classical monocytes and intermediate/non-classical monocytes. (**F**) Concentrations of cytokines and chemokines. Graphs show medians with standard deviations. DCs: dendritic cells, Lin+: lineage (i.e., CD3, CD19 or CD56)-positive. * Values above the upper limit of quantification (IL-1RA at 3 and 4 h, panel (**F**)).

**Figure 2:**
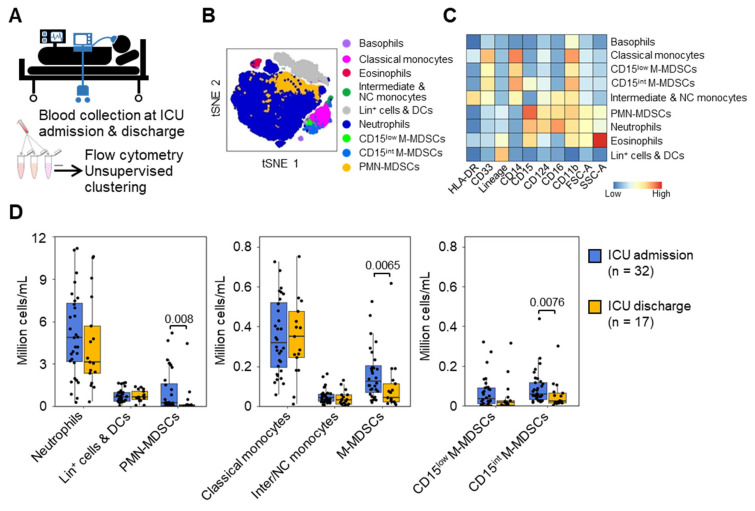
MDSC-like cells in the blood of mechanically ventilated ICU patients. (**A**) Study design. Blood was collected at ICU admission (n = 32) and at ICU discharge (n = 17) from mechanically ventilated ICU patients without infection and analyzed as described in Figure 1. (**B**) t-SNE plot of leukocyte populations. (**C**) Relative expression levels of cell surface markers and forward and side scatter areas (FSC-A, SSC-A) of leukocyte populations. (**D**) Absolute counts (millions of cells per mL) of neutrophils, lineage-positive (lin+) cells and dendritic cells (DCs), polymorphonuclear MDSCs (PMN-MDSCs), classical monocytes, intermediate/non-classical (inter/NC) monocytes, monocytic MDSCs (M-MDSCs), and CD15^low^ and CD15^intermediate^ (CD15^int^) M-MDSCs. Boxplots show medians and upper and lower quartiles, whiskers show the 5 to 95 percentiles, and dots show the individual values.

One hundred μL of blood was added to DURAClone tubes (Beckman Coulter, Brea, CA, USA) containing lyophilized antibodies directed against (clone name, labelling) HLA-DR (Immu-357, FITC), CD3 (UCHT1, APC-AF700 or AF-700), CD11b (Bear1, PE-Cy7), CD14 (RMO52, APC-AF750), CD15 (80H5, Pacific Blue), CD16 (3G8, ECD), CD19 (J3-119, APC-AF700 or AF-700), CD33 (D3HL60.251, APC), CD45 (J33, Krome Orange), CD56 (NKH-1, APC-AF700 or AF-700), and CD124 (G077F6, PE). Lineage-positive (lin+) cells were defined as positive for either CD3, CD19 or CD56. After 20 min at 22 °C in the dark, 900 μL of 1 × BD FACS™ lysing solution (BD Biosciences, San Jose, CA, USA) was added to lyse the red blood cells and fix leukocytes. The tubes were kept at −80 °C until analysis. The samples were thawed 1 min at 37 °C, washed and reconstituted with PBS containing 0.5% BSA and sodium azide 0.02%, and analyzed on an Attune NxT Flow Cytometer (Thermo Fisher scientific, Waltham, MA, USA). Debris, doublets, and non-hematopoietic cells were excluded using FlowJo™ 10.6.2 (BD Life Sciences, Ashland, OR, USA) (Supplementary Figure S1). We performed unsupervised clustering with FlowSOM using the biexponential transformed and normalized expression levels of the markers and relative forward and side scatter areas (FSC-A, SSC-A). The resulting 100 clusters were reduced to 30 metaclusters and manually merged into populations based on marker expression and biological knowledge: MDSCs, basophils, eosinophils, neutrophils, classical monocytes, intermediate/non-classical (NC) monocytes, lineage-positive (lin^+^) cells, and DCs (Figure 1A,B and 2A) [32]. PMN-MDSCs (CD11b^+^ CD14^−^ CD15^+^ CD16^+^ CD33^−^ HLA-DR^−^ cells) were identified based on the low expression levels of CD11b and CD16 when compared to mature PMNs. M-MDSCs (CD11b^+^ CD14^+^ CD15^−/low^ CD16^−^ CD33^+^ HLA-DR^−/low^ cells) were identified based on the low expression levels of HLA-DR [11,13,23,33]. Unsupervised clustering identified CD15^low^ and CD15^int^ M-MDSC subpopulations in blood samples from the PIPOVAP study.

### 2.3. Measurement of Serum Mediators by Multiplex Bead Assay

The concentrations of IL-1RA, IL-6, IL-8, IL-10, CCL2, CCL3, CCL4, and TNF in the serum from healthy volunteers challenged with LPS were quantified using Luminex technology (Luminex Corporation, Austin, TX, USA). The concentrations of 45 mediators in the serum samples obtained from patients at their admission to the ICU (PIPOVAP study) were determined by the clinical laboratory of the Service of Immunology and Allergy, Lausanne University Hospital, using Luminex xMAP Technology as described [34].

### 2.4. Statistical Analysis

The data generated during this study, as well as detailed patient’s demographics, are available in Supplementary Table S1. Baseline comparisons were performed using the chi-square exact test, Mann–Whitney U test, and Kruskal–Wallis test, as appropriate. The relationship between the percentages of cell population and clinical data (i.e., categorial variables) was assessed using the Mann–Whitney U test. The expression levels of PMN-MDSCs ≤ 10% and >10% of leukocytes and of CD15^int^ M-MDSCs ≤ 1.3% and >1.3% of leukocytes (cutoff value based on tertile) were categorized as high and low levels and were used to analyze mortality over time. Survival curves were compared using the log-rank test. Luminex data were analyzed using Spearman’s rank correlation controlling for FDR (false discovery rate) using the Benjamini and Hochberg procedure. Mediators with a coefficient of correlation greater than 0.3 with a population of MDSCs are reported. Statistics and illustrations were achieved employing R v.3.6.0 (R Foundation for Statistical Computing, Vienna, Austria). Each dot represents an individual sample. *, *p* < 0.05; **, *p* ≤ 0.01; ***, *p* ≤ 0.001; ****, *p* ≤ 0.0001.

## 3. Results

### 3.1. MDSC-like Cells in Healthy Subjects Challenged with Endotoxin

We used an experimental model of endotoxemia to delineate the impact of systemic inflammation on the expression of MDSCs since endotoxemia is a common feature of ICU patients. Eight healthy male volunteers (median age 23.5 years, interquartile range [IQR]: [22,23,24,25,26,27]) were challenged with 2 ng/kg of LPS (Table 1). Blood was collected before and up to 336 h after endotoxin administration (Figure 1A).

The baseline leukocyte count was 4.7 [4.5–5.6] × 10^6^ cells/mL. Leukocytes dropped 2.3-fold 1 h after the endotoxin challenge, increased above normal values over the next 8 h, and returned to baseline values within 24 to 168 h (Supplementary Figure S2). We then analyzed blood leukocytes using a flow cytometry pipeline constructed to minimize technical and analytical variations. Whole blood was collected in tubes containing lyophilized antibodies, the samples were frozen to perform all acquisitions at once, and the data were analyzed via unsupervised clustering (see Section 2.2 and [23,33]). Unsupervised clustering identified cell populations phenotypically reminiscent of PMN-MDSCs and M-MDSCs alongside basophils, eosinophils, neutrophils, classical monocytes, intermediate/non-classical (NC) monocytes, and lineage-positive (lin^+^: CD3, CD19 or CD56 positive) cells and DCs (Figure 1B,C). For the sake of clarity, we will refer to PMN-MDSCs and M-MDSCs in the next chapters, although the immunosuppressive activity of the cells was not characterized functionally.

Neutrophils declined from 2.5 [2.2–3.1] × 10^6^ cells/mL at baseline to 0.79 cells [0.58–1.23] × 10^6^ cells/mL 1 h after endotoxin challenge (*p* = 0.007). They increased between 2 and 8 h and returned to baseline levels after 168 h (Figure 1D). Neutrophils constituted 40–69% of leukocytes throughout follow-up. At baseline, the number of PMN-MDSCs was 0.03 [0.02–0.07] × 10^6^ cells/mL, representing 0.6% [0.4–1.4] of leukocytes (Figure 1D). PMN-MDSCs increased 2 h after endotoxin challenge and plateaued at 3.7 [2.6–4.2] × 10^6^ cells/mL after 6 h (0 versus 6 h: *p* = 0.0002), where they represented up to 30% [20–36] of leukocytes. PMN-MDSCs returned to baseline levels after 24 h (Figure 1D).

Monocytes (classical, intermediate, and non-classical) dropped from 0.32 [0.26–0.35] × 10^6^ cells/mL at baseline to 0.036 [0.025–0.041] × 10^6^ cells/mL 1 h after endotoxin challenge (*p* = 0.008). Monocytes steadily increased to baseline levels after 8 h (0.31 [0.22–0.39] 10^6^ cells/mL, *p* = 0.20 versus baseline). Like PMN-MDSCs, M-MDSCs were lowly abundant at baseline (0.026 [0.022–0.079] × 10^6^ cells/mL), representing 0.6% [0.4–1.6] of leukocytes (Figure 1E). M-MDSCs increased between 4 and 8 h to reach 0.22 [0.18–0.29] × 10^6^ cells/mL (0 versus 8 h: *p* = 0.0006). M-MDSC levels returned to baseline after 24 h. As a result, M-MDSCs represented 8.2% [5.9–27.3] of monocytic cells at homeostasis, but 46.5% [31.7–54.3], 43.5% [39.8–49.8] and 46.9% [30.2–57.4] of monocytic cells 4, 6 and 8 h after endotoxin challenge (Figure 1E).

Since cytokines influence the production of MDSCs [12,16,17], we measured the concentrations of IL-1RA, IL-6, IL-8, IL-10, CCL2, CCL3, CCL4, and TNF in blood samples. As expected, TNF peaked after 1 h, while IL-6, IL-8, CCL2, CCL3, CCL4 reached their maximum values after 2 h, and IL-1-RA and IL-10 reached their maximum after 3 h of endotoxin challenge (Figure 1F). So, proinflammatory cytokine response preceded MDSC accumulation in peripheral blood. The cytokines returned to baseline levels after 4–8 h, except for CCL2 and IL-1RA, which returned to baseline levels between 8 and 336 h. Overall, endotoxin administration induced a massive and transient accumulation of MDSC-like cells in the circulation, accompanied by elevated levels of cytokines.

### 3.2. MDSC-like Cells in Mechanically Ventilated ICU Patients

We set up a clinical study to test whether MDSC-like cells represent useful biomarkers in critically ill patients with a high probability of being infected during hospitalization. Thirty-three uninfected mechanically ventilated ICU patients (median age 65 [52–68] years) were studied (Figure 2A). The median ICU stay was 8.0 [6.0–14.0] days. Twenty-five patients (75.7%) developed an infection during their ICU stay (time to onset: 1 [0–2] day), consisting of ventilator–associated pneumonia (VAP) in fifteen patients (45.4%) and a non-VAP in ten patients (30.3%). Four patients (12.5%) died (days till death: 7.0 [5.5–9.3] days) (Table 1). Survivors and non-survivors had similar acute physiology and chronic health evaluation II (APACHE II) and sequential organ failure assessment (SOFA) scores (Table 1). The CRP values were high in ICU patients when compared to healthy subjects (normal values: <5 mg/L). In contrast, lactate levels in patients were close to normal values (<1 mol/L), suggesting that patients were not in acute circulatory distress/shock. Indeed, patients were selected without infection and with a likelihood of surviving the first 24 h. The blood was collected at the time of ICU admission in all patients and at ICU discharge in 17 patients. Flow cytometry analyses identified basophils, eosinophils, neutrophils, classical monocytes, intermediate/non-classical monocytes, lin^+^ cells and DCs, as well as cell populations phenotypically reminiscent of PMN-MDSCs and M-MDSCs, which were further sub-clustered into CD15 intermediate (CD15^int^) and CD15^low^ M-MDSCs (Figure 2B,C). Counts and frequencies of neutrophils and monocytes did not differ between admission and discharge from the ICU (Figure 2D and Supplementary Figure S3).

PMN-MDSCs decreased from 0.24 [0.05–1.6] × 10^6^ cells/mL at ICU admission to 0.05 [0.01–0.13] × 10^6^ cells/mL at discharge (*p* = 0.008), while M-MDSCs decreased from 0.13 [0.07–0.21] to 0.04 [0.02–0.11] × 10^6^ cells/mL (*p* = 0.007) (Figure 2D and Supplementary Figure S3). The results were similar when the analyses were performed on paired samples from survivors. Among M-MDSCs, CD15^int^ M-MDSCs were more abundant than CD15^low^ M-MDSCs at ICU admission (0.059 versus 0.036 × 10^6^ cells/mL, *p* > 0.05). Both populations decreased at discharge (0.024 versus 0.018 × 10^6^ cells/mL).

PMN-MDSC and M-MDSC numbers at ICU admission were significantly higher than those measured in healthy subjects before the endotoxin challenge (12 and 6.5-fold, *p* = 0.0016 and *p* = 0.00016), indicating that the deterioration in underlying conditions was associated with higher levels of MDSCs. Yet, PMN-MDSCs at ICU admission were 9.1-fold lower than 8 h after endotoxin challenge (*p* = 0.019), while M-MDSC counts were comparable under the same conditions. This confirmed the power of LPS to stimulate the rise of MDSCs, particularly PMN-MDSCs. PMN-MDSC and M-MDSC levels at ICU discharge were comparable to those of healthy subjects (*p* = 0.17 and *p* = 0.67) (Supplementary Figure S4A,B).

### 3.3. MDSC-like Cells in Relation with Cytokines and Growth Factors Levels in ICU Patients

**Figure 3:**
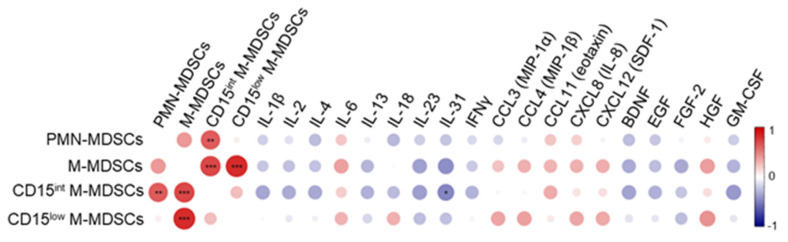
Correlation matrix between MDSC-like cells, cytokines, chemokines and growth factors. Blood was collected from patients on admission to the ICU to quantify cytokines, chemokines and growth factors (see Section 2). Correlations were calculated using Spearman’s rank correlation, which was controlled for false discovery rate using the Benjamini and Hochberg step-up procedure. The correlation plot represents mediators with a correlation coefficient ≥ 0.3 or ≤ −0.3 with at least one population of MDSCs. * *p* < 0.05, ** *p* ≤ 0.01, *** *p* ≤ 0.001. The color scale ranges from blue for negative correlation to red for positive one. The size of the dots is proportional to the *p* value.

We performed correlation studies between cytokines/growth factors and MDSCs in blood collected at ICU admission (Figure 3). We quantified 23 cytokines, 10 chemokines, and 12 growth factors. A coefficient of correlation (ρ) ≥ 0.3 or ≤ −0.3 with MDSCs was detected for 19 mediators (ρ ≥ 0.3 for IL-6, IL-18, CCL3, CCL4, CCL11, CXCL8, CXCL12, HGF and ρ ≤ −0.3 for IL-1β, IL-2, IL-4, IL-13, IL-23, IL-31, IFNγ, BDNF, EGF, FGF-2, GM-CSF). The frequencies of PMN-MDSCs, M-MDSCs and CD15^int^ M-MDSCs correlated negatively with IL-18 and BDNF, IL-31 and BDNF, IL-4, IL-31, IFNγ, BDNF, EGF, and GM-CSF, while the frequencies of M-MDSCs and CD15^low^ M-MDSCs correlated positively with IL-6, CXCL8 and HGF and IL-6, IL-18, CXCL8, and HGF, respectively. After false discovery rate correction, the negative correlation between IL-31, a member of the IL-6 cytokine family, and CD15^int^ M-MDSCs remained statistically significant (ρ = −0.48, *p* = 0.049). This negative correlation is consistent with the observation that IL-31 inhibits the motility and activity of MDSCs in a model of breast carcinoma [35]. Finally, PMN-MDSC and CD15^int^ M-MDSC expression levels correlated positively (ρ = 0.43, *p* = 0.03).

### 3.4. MDSC-like Cells and Nosocomial Infections in ICU Patients

**Figure 4:**
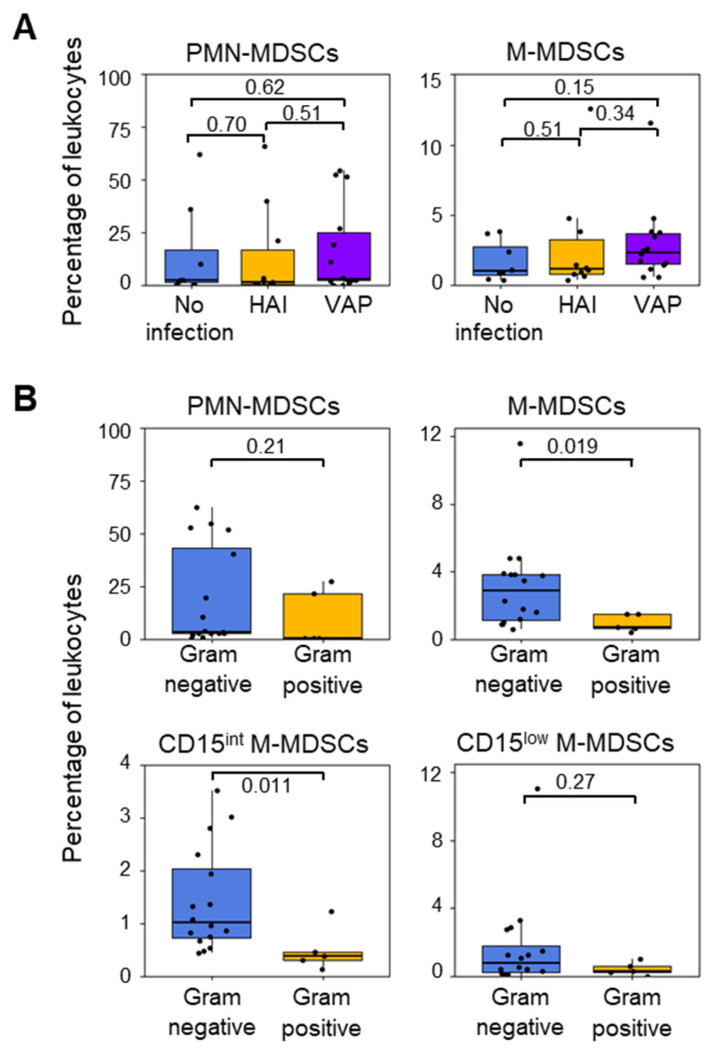
Proportions of MDSC-like cells in patients who developed or did not develop infections during their ICU stay. (**A**) PMN-MDSCs and M-MDSCs in patients who did (n = 25) or did not (n = 8) develop an infection. Infections were sorted into hospital-acquired infection (HAI, n = 10) and ventilator-associated pneumonia (VAP, n = 15). (**B**) PMN-MDSCs, M-MDSCs, CD15int M-MDSCs, and CD15low M-MDSCs at ICU admission in patients who subsequently developed Gram-negative or Gram-positive bacterial infections. Boxplots show medians and upper and lower quartiles, whiskers show 5 to 95 percentiles, and dots show individual values to the *p* value.

PMN-MDSCs and M-MDSCs at ICU admission (percentages and absolute counts) were not related to the development of hospital-acquired infection (HAI) and VAP (Figure 4A and Supplementary Figure S5A). Neither were they associated with the time to develop infection and the occurrence of sepsis or septic shock. The absence of an association between MDSCs and infection could reflect that MDSCs were elevated prior to ICU admission, while the time to develop infection was short. Furthermore, no other cell population (basophils, eosinophils, neutrophils, monocytes, monocytes, and lin^+^ cells and DCs) was related to the development of infections.

Eighteen patients developed a Gram-negative bacterial infection, and four patients developed a Gram-positive bacterial infection. The frequency (% of total leukocytes) and the absolute count of PMN-MDSCs at ICU admission were not related to infection with either Gram-positive or Gram-negative bacteria (Figure 4B and Supplementary Figure S5B). In contrast, the levels of M-MDSCs at ICU admission were four times higher in patients who developed Gram-negative bacterial infections (2.8% [1.1–3.8]) than in patients who developed Gram-positive bacterial infections (0.7% [0.6–1.5]) (*p* = 0.019). Furthermore, significant differences in the abundance of CD15^int^ M-MDSCs were found between patients who developed infections with Gram-negative and Gram-positive bacteria (Gram-negative: 1.0% [0.7–2.0] or 0.095 [0.048–0.146] × 10^6^ cells/mL; Gram-positive: 0.4% [0.3–0.5] or 0.027 [0.025–0.0.034] × 10^6^ cells/mL for; *p* = 0.011 and *p* = 0.04) (Figure 4B).

### 3.5. MDSC-like Cells and Outcome of ICU Patients

**Figure 5:**
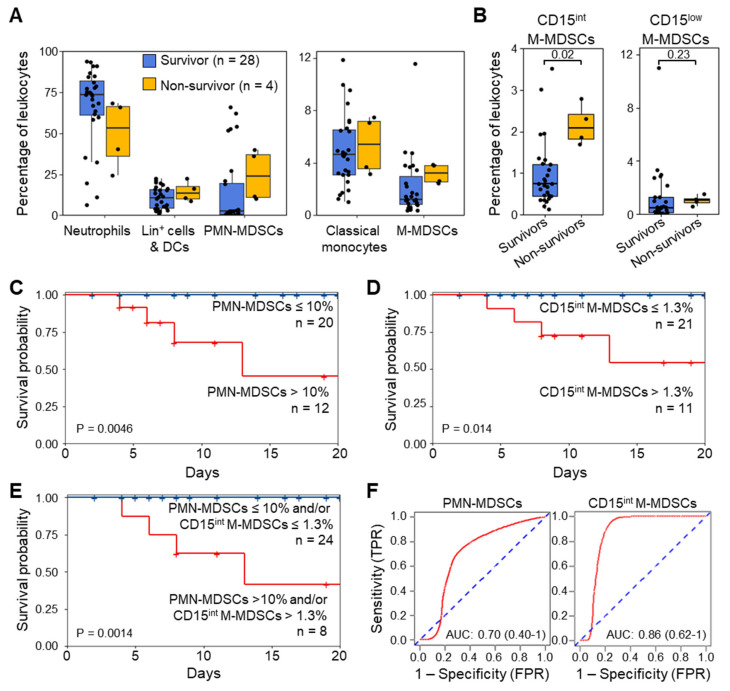
MDSC-like cells at ICU admission in relation to patient’s outcome. (**A**) Leukocyte populations in ICU survivors (n = 28) and non-survivors (n = 4). (**B**) CD15^int^ and CD15^low^ MDSCs in survivors and non-survivors. Boxplots show medians and upper and lower quartiles, whiskers 5 to 95 percentiles, and dots individual values. The association of MDSCs with survival was assessed using the Mann–Whitney U and the Kruskal–Wallis tests. (**C**) Kaplan–Meier survival curves based on low and high levels of PMN-MDSCs (≤ 10% and >10% of leukocytes). (**D**) Kaplan–Meier survival curves based on low and high levels of CD15^int^ M-MDSCs (≤1.3% and >1.3% of leukocytes). (**E**) Kaplan–Meier survival curves based on low and high levels of PMN-MDSCs and/or CD15^int^ M-MDSCs. (**F**) Receiver operating characteristic (ROC) curves of PMN-MDSCs and CD15^int^ M-MDSCs for mortality. The cutoff values used to segregate low and high levels of PMN-MDSCs and CD15^int^ M-MDSCs were based on the highest tertile. Statistical differences were assessed using the log-rank test. AUC: area under the curve; CI: confidence of intervals; FPR: false positive rate; TPR: true positive rate.

The abundance of PMN-MDSCs and M-MDSCs at ICU admission were higher in patients who died during their hospital stay (PMN-MDSCs and M-MDSCs: 1.18 [0.74–1.92] and 0.19 [0.17–0.22] × 10^6^ cells/mL; 23.9% [11.1–37.4] and 3.2% [2.6–3.8] of leukocytes, respectively), although the results were statistically significant only for CD15^int^ M-MDSCs (survivors: 0.05 [0.03–0.10] × 10^6^ cells/mL, 0.7% [0.5–1.3], non-survivors: 0.12 [0.10–0.14] × 10^6^ cells/mL, 2.1% [1.8–2.4], *p* = 0.02) (Figure 5A,B).

To test whether high levels of MDSCs were associated with worse outcomes, we stratified patients according to low and high expression levels of MDSCs using cutoff values corresponding to the highest tertile (≤ 10% and > 10% for PMN-MDSCs, ≤1.3% and >1.3% for CD15^int^ M-MDSCs). All patients with a low abundance of PMN-MDSCs (n = 20) survived, while 33% of patients with high levels of PMN-MDSCs (n = 12) died (*p* = 0.0046) (Figure 5C). Additionally, patients with low levels of CD15^int^ M-MDSCs (n = 21) survived, while 36% of patients with high levels of CD15^int^ M-MDSCs (n = 11) died (*p* = 0.014, Figure 5D). All patients with low levels of PMN-MDSCs and/or CD15^int^ M-MDSCs (n = 24) survived, while 50% of patients with high levels of PMN-MDSCs and/or high levels of CD15^int^ M-MDSCs (n = 8) died (*p* = 0.0014) (Figure 5E). Receiver operating characteristic (ROC) curve analyses were performed to evaluate the predictive survival performance of MDSCs (Figure 5F). The area under the ROC curve (AUC) for PMN-MDSCs was 0.70 (95% confidence interval [CI] = 0.40–1). The AUC for CD15^int^ M-MDSCs was 0.86 (95% CI = 0.62–1).

## 4. Discussion

We report that MDSC-like cells rise sharply and transiently in the blood of healthy subjects challenged with endotoxin. In uninfected mechanically ventilated patients admitted to the ICU, the levels of CD15^int^ M-MDSCs correlated with the development of Gram-negative bacterial infections, whereas elevated levels of PMN-MDSCs or CD15^int^ M-MDSCs were associated with mortality. Therefore, MDSCs (or MDSC-like cells) may represent biomarkers for critically ill and infected patients [15,36].

The abundance of MDSCs in healthy subjects and in sepsis patients differed noticeably between studies [15,16,19,20,21,23,36,37,38,39,40]. This is partly because the quantification of MDSCs via flow cytometry is influenced by factors including the type of sample (whole blood, freshly isolated or frozen PBMCs) and the immunophenotyping strategy [13,15,23,36,40]. It is also possible that MDSCs progress phenotypically during sepsis. In our study, we used phenotypical standards [13]. To minimize analytical variations, we collected whole blood in tubes containing lyophilized antibodies and used clustering of flow cytometry data to identify MDSCs. An advantage of whole blood is that it enables MDSCs and neutrophils to be quantified in a single sample. However, this is at the expense of using the low-density characteristic of PMN-MDSCs for discrimination purposes. While functional suppressive activity is the gold standard for defining MDSCs [11,12,17], phenotypical evaluation via flow cytometry is preferred in routine practice, and more so on whole blood than on density gradient-purified PBMCs. To improve immunophenotyping, the panel of antibodies could be enriched. For example, we could target lectin-type oxidized LDL receptor 1, which is expressed by human PMN-MDSCs in cancer patients and septic shock and severe coronavirus disease 2019 (COVID-19) patients [41,42]. Another candidate is CD300ld, which was discovered in an in vivo CRISPR-Cas9 screen and was shown to be increased in PMN-MDSCs involved in tumor progression in mouse models, as well as in human tissues from colon and lung cancers [43].

In sepsis patients, single-cell RNAseq analyses of MDSCs have shown specific transcriptomes [22] but have failed to devise phenotypic markers, allowing for the unambiguous identification of MDSCs via flow cytometry. Furthermore, in-depth explorations of neutrophilic cells (i.e., including cells that should be PMN-MDSCs) via single-cell RNAseq suggested that neutrophils transit through states rather than durable subsets that are more easily traceable and that functional signatures transcend specific subsets of activation [44,45]. A recent whole-blood single-cell multiomic atlas in sepsis patients identified immunosuppressive CD66b^+^ neutrophils and features of emergency granulopoiesis with a higher frequency of immature neutrophils in a subgroup of patients with poor outcomes [46]. Altogether, the most recent single-cell analyses indicate that it may be difficult to identify universal, stably expressed, phenotypical markers to track MDSCs.

Human experimental endotoxemia is the only model available to study the impact of a systemic inflammatory response in humans in a well-controlled setting. M-MDSCs showed an initial drop before a progressive increase, which reflects the transient monocytopenia observed in endotoxemia [47]. Even considering this initial decrease, globally, MDSCs (re)increased quite rapidly after the LPS challenge. This is in line with mouse studies showing that MDSCs increased in blood, spleen, and liver 3 to 12 h after intraperitoneal or the intratracheal administration of LPS [48,49,50]. In models of chronic sepsis induced by cecal ligation and puncture, MDSCs increased more gradually over several days [51,52], suggesting that the inflammatory burst has a deep impact on the generation of MDSCs. Indeed, the administration of LPS increased cytokines and chemokines that stimulate myelopoiesis and the development of MDSCs. Endotoxemia is common in ICU patients suffering from trauma, abdominal and cardiovascular surgery, as well as COVID-19 and bacterial sepsis [26,53]. Thus, endotoxemia may contribute to an increase in MDSCs in uninfected and infected critically ill patients.

Our analytical strategy identified CD15^int^ and CD15^low^ M-MDSCs subgroups in ICU patients. CD15, known as Lewis X antigen, is expressed by granulocytes but also monocytes, macrophages, eosinophils, mast cells, and myeloid precursor cells. Interestingly, a study in the late 1990s described a subpopulation of whole-blood monocytes that resembles M-MDSCs with high SSC parameters and the robust expression of CD15 and ROS [54]. Clustering did not identify the CD15^int^ M-MDSCs subpopulation in the endotoxin study, possibly due to the limited number of individuals analyzed or because this subpopulation is expressed more specifically in critically ill patients. Indeed, two recent studies applying unsupervised clustering of PBMCs and semi-automated analysis of whole blood in sepsis patients reported a dichotomous distribution of CD15 expression by what appears to be M-MDSCs [15,36]. A so-called unconventional CD15^+^ CD11b^+^ CD14^+^ CD33^+^ CD66b^+^ HLA-DR^−^ subset increased early during sepsis and returned to physiological levels in survivors [15]. Likewise, we observed that CD15^int^ M-MDSCs were elevated at ICU admission compared to ICU discharge. The subdivision of M-MDSCs based on CD15 might be useful in stratifying critically care patients. Unfortunately, due to the design of our study, we were not able to cell sort and further characterize this subpopulation of M-MDSCs.

MDSCs increase in patients was probably initiated before their admission to the ICU. If so, we possibly did not capture the full range of mediators relevant to MDSC expansion in the blood collected at ICU admission. It would explain why only one association was statistically significant. The sample size combined with the large number of comparisons (45 mediators and four cell populations) is another factor that reduced sensitivity. Though not statistically significant after correction for multiple testing, positive associations involving IL6, CCL3, CCL4, CCL11, CXCL12, and HGF would be consistent with the impact these mediators have on the generation of MDSCs. For example, IL-6 plays a critical role in regulating the accumulation and activation of MDSCs. Moreover, MDSCs have been reported to express receptors for IL-6 (IL-6R), CCL3, CCL4, CCL11 (CCR2/3/5), CXCL8 (CXCR1/2), CXCL12 (CXCR4/7), and HGF (HGFR/Met receptor). Unfortunately, we were not able to quantify the expression of these receptors.

MDSC levels did not correlate with nosocomial infections, which contrasts with other reports [19,20,21,23]. However, high levels of M-MDSCs, particularly CD15^int^ MDSCs, correlated with the occurrence of Gram-negative bacterial infection. Additionally, M-MDSCs increased 10-fold during endotoxemia, and high levels of M-MDSCs have been associated with Gram-negative bacterial sepsis [19,37]. Of note, on a per-cell basis, M-MDSCs are more potent immunosuppressive than PMN-MDSCs. Hence, a minor subpopulation of MDSCs may have a significant pathophysiological impact. Remarkably, patients who did not survive presented to the ICU with high levels of PMN-MDSCs and CD15^int^ M-MDSCs, while clinical severity scores were slightly lower compared to survivors. The stratification of the patients into those with high and low expression of MDSCs and ROC curve analyses revealed a good discriminative value of MDSCs. Accordingly, high levels of PMN-MDSCs and/or M-MDSCs at study inclusion correlated with mortality in patients with sepsis and COVID-19 and in non-surgical ICU patients. In addition, MDSCs play a role in establishing or maintaining a protracted immunosuppressive environment, contributing to chronic critical illness, secondary infections, and long-term morbidity and mortality in ICU patients [16,19,20,21,22,33,39]. Overall, the data suggest that MDSCs could be biomarkers for stratifying patients and selecting those who might benefit from targeted therapy. Preclinical studies have shown promising results with strategies to reduce MDSCs levels and increase T-cell function during sepsis, while phase II clinical trials targeting MDSCs in oncologic patients are in progress.

Our study has several limitations. We identified MDSCs through phenotypical and non-functional analyses. However, the immunosuppressive function of MDSCs isolated based on the expression of cell surface markers (through magnetic or flow cytometry cell sorting) has been reported in numerous studies [19,38]. Of note, quantification of MDSC-like cells in blood via flow cytometry is currently the best option for clinical development, while it is not conceivable to introduce functional tests for immunosuppressive functions of MDSCs in routine. We used a stain/fix/freeze procedure before samples were analyzed. Although unusual, this method, developed by others (see, for example, [55,56]), permits the acquisition of all samples in a row, thus limiting the impact of possible fluctuations in instrument performance. The endotoxemia study was performed with males. The harmonization of the study group was intended, but it may be considered suboptimal as there is sexual dimorphism in the host response to microbial products. One should keep in mind that MDSCs are barely detected in the blood of healthy subjects. Therefore, it might be problematic to define a normal range in healthy people and challenging to stratify via flow cytometry ICU patients for targeted therapy. The limited sample size and the quantification of mediators at a given time point restricted the sensitivity of detecting associations between MDSCs and biological parameters in ICU patients. In addition, the predictive model of outcomes is based on a small number of patients who did not survive. Thus, larger cohorts should be studied to confirm that MDSC levels might be used to stratify patients, and to validate the association between elevated levels of PMN-MDSCs and/or CD15^int^ M-MDSCs with mortality and to characterize the functions of CD15^int^ M-MDSCs.

## 5. Conclusions

In conclusion, we show that MDSC-like cells were highly responsive to endotoxin challenge and that elevated levels of PMN-MDSCs and CD15^int^ M-MDSCs in blood correlated with the development of Gram-negative bacterial nosocomial infections and patient outcomes. Our observations support the idea that MDSCs may be used as biomarkers contributing to mortality prognosis and risk stratification of ICU patients for targeted therapy.

## Figures and Tables

**Table 1:** Characteristic of healthy volunteers and patients.

	Endotoxin Study	ICU Study	
	Baseline	ICU Survivor	ICU Non-Survivor
Number of subjects/patients	8	29	4
Gender, male	8 (100%) *	15 (52%)	3 (75%)
Age (years)	23.5 [22–27] *	65 [52–68]	63 [53–67]
Severity of illness at admission:			
Mechanical ventilation	-	29 (100%)	4 (100%)
APACHE II score	-	19 [16–23]	20.5 [18.5–22.3]
SOFA score	-	12 [10–14]	11.5 [7.8–15.5]
Developed a secondary infection	-	23 (79%)	2 (50%)
Type of secondary infection:			
VAP/HAP	-	14 (48%)	1 (25%)
HAI	-	9 (31%)	1 (25%)
ICU stay (days)	-	8.5 [6.00–16.25]	7 [5.50–9.25]
Leukocytes (× 10^9^/L)	4.7 [4.5–5.6]	8.4 [5.6–9.8]	6.0 [4.7–7.3]
CRP (mg/L)	-	168 [122–281]	103, 86 ^†^
Lactate (mmol/L)	-	1.1 [0.8–1.7]	0.8, 0.6 ^†^

* Medians [IQR] or n (%). ^†^ Data available from 2 patients. APACHE II: acute physiology and chronic health evaluation II; CRP: C-reactive protein; HAI: hospital-acquired pneumoniae; HAP: hospital-acquired pneumonia; ICU: intensive care unit; SOFA: sequential organ failure assessment; VAP: ventilator-associated pneumonia; 95% CI: 95% confidence interval.

## Data Availability

All data generated or analyzed during this study are included in this article and its supplementary information files. Restrictions apply to patient’s characteristics due to privacy or ethical restrictions.

## Supplementary Materials

The following supporting information can be downloaded at: https://www.mdpi.com/article/10.3390/cells13040314/s1, Figure S1: Gating strategy of flow cytometry data to exclude debris, doublets and CD45- non-hematopoietic cells (**A**), univariate histogram plots of cell surface marker expression by whole blood cells from the endotoxemia study (**B**) and ICU study (**C**) study, and back-gating of samples from ICU study (**D**); Figure S2: Leukocyte counts in the blood of healthy subjects infused with endotoxin; Figure S3: MDSC-like cells in non-infectious mechanically ventilated ICU patients; Figure S4: Comparison of MDSC-like cells levels between healthy subjects challenged with endotoxin and ICU patients; Figure S5: Absolute counts of MDSC-like cells in relation with the development of infection in mechanically ventilated ICU patients; Table S1: Data generated during this study. Table S1 contains all data and analyses, as well as detailed patient’s demographics. Pages 1–4 are related to the endotoxin study, while pages 5–8 are related to the PIPOVAP study.
