# Supplementary material for: Myeloid-Derived Suppressor-like Cells as a Prognostic Marker in Critically Ill Patients: Insights from Experimental Endotoxemia and Intensive Care Patients
Source: Cells. 2024 Feb 8;13(4):314. doi: 10.3390/cells13040314 (PMC10887109; doi:10.3390/cells13040314)
Supplement: Supplementary file 1 [file cells-13-00314-s001.zip › cells-2709553-supplementary.pdf]

Myeloid-derived suppressor-like cells as a prognostic marker in critically ill patients: insights from experimental endotoxemia and intensive care patients

Irene T. Schrijver [1†], Jacobus Herderschee [1†], Charlotte Theroude [1], Antonios Kritikos [1], Guus Leijte [2,3], Didier Le Roy [1], Maelick Brochut [1], Jean-Daniel Chiche [4], Matthieu Perreau [5], Giuseppe Pantaleo [5], Benoit Guery [1], Matthijs Kox [2,3], Peter Pickkers [2,3], Thierry Calandra [1‡], Thierry Roger [1,*]

1 Infectious Diseases Service, Department of Medicine, Lausanne University Hospital and University of Lausanne, Lausanne, Switzerland
2 Radboud Center for Infectious Diseases, Radboud University Medical Center, Nijmegen, The Netherlands
3 Department of Intensive Care Medicine, Radboud University Medical Center, Nijmegen, The Netherlands
4 Service of Adult Intensive Care Medicine, Lausanne University Hospital and University of Lausanne, Lausanne, Switzerland
5 Service of Immunology and Allergy, Department of Medicine, Lausanne University Hospital and University of Lausanne, Lausanne, Switzerland
* Correspondence: Thierry.Roger@chuv.ch; Tel.: +41-21-314-1038; ORCID 0000-0002-9358-0109
† Co-authorship

19 Supplementary Figures

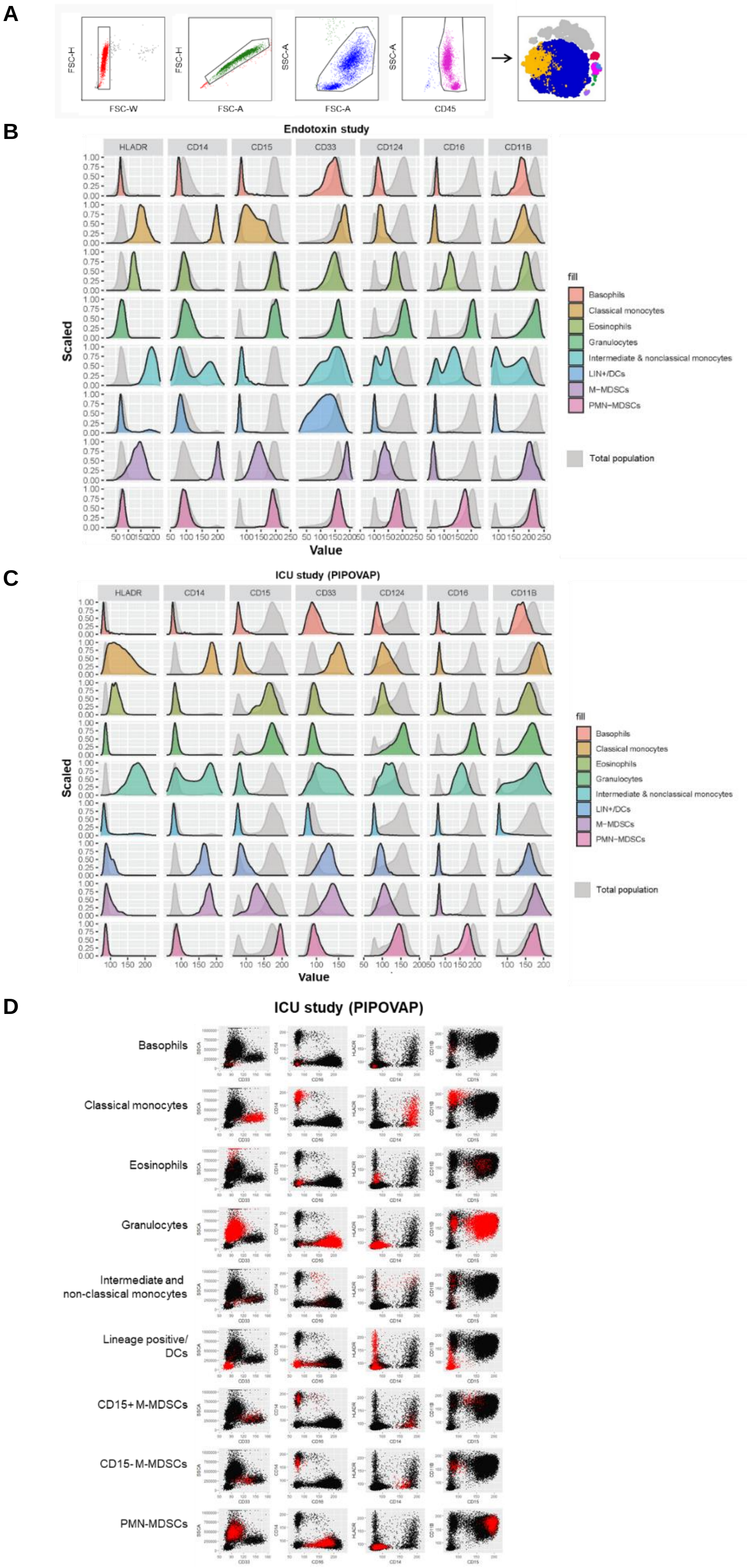

Figure S1. Gating strategy of flow cytometry data to exclude debris, doublets and CD45- non-hematopoietic cells (**A**), univariate histogram plots of cell surface marker expression by whole blood cells from the endotoxemia study (**B**) and ICU study (**C**) study, and back-gating of samples from ICU study (**D**).

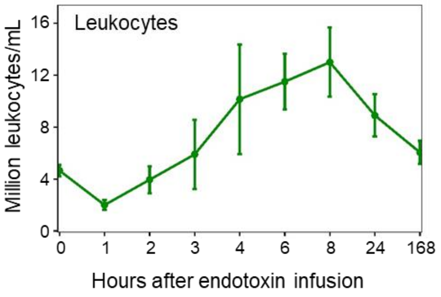

Hours after endotoxin infusion	Million leukocytes/mL (median)	IQR
0	4.7	4.5-5.6
1	2.0	1.9-2.4
2	4.0	3.9-5.1
3	5.9	4.7-7.7
4	10.2	7.2-12.7
6	11.5	10.4-16.1
8	13.0	12.5-17.6
24	8.9	8.0-11.0
168	6.1	5.6-6.5

Figure S2. Leukocyte counts in the blood of healthy subjects infused with endotoxin. Eight healthy subjects were infused with 2 ng/kg endotoxin. Blood was collected just before (0) and 1, 2, 3, 4, 6, 8, 24 and 168 hours after endotoxin infusion. $CD45^+$ cells were quantified by flow cytometry as described in *Materials and Methods*, and data expressed as millions of leukocytes/mL of blood. **Left**) The graph shows medians with standard deviations. **Right**) Data are medians and interquartile ranges (IQR).

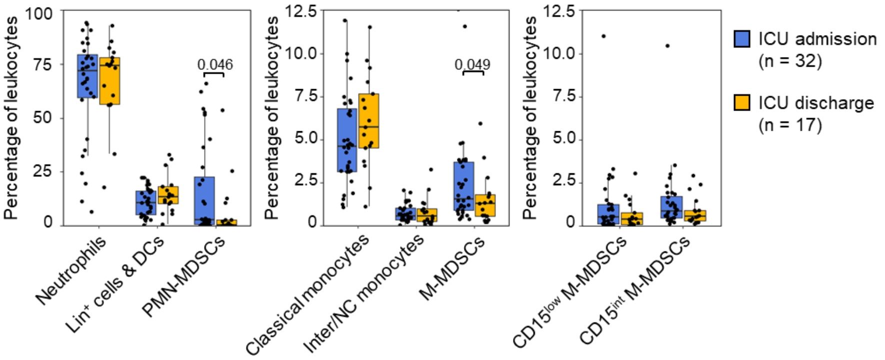

Figure S3. MDSC-like cells in non-infectious mechanically ventilated ICU patients. Blood was collected at ICU admission (n = 32) and at ICU discharge (n = 17) from mechanically ventilated ICU patients without infection and analyzed by flow cytometry and unsupervised clustering as described in **Figure 1**. Data are expressed as the percentage in leukocytes of neutrophils, lineage positive (lin+) cells and dendritic cells (DCs), polymorphonuclear MDSCs (PMN-MDSCs), classical monocytes, intermediate/non-classical (inter/NC) monocytes, monocytic MDSCs (M-MDSCs), and $CD15^{low}$ and $CD15^{intermediate}$ ($CD15^{int}$) M-MDSCs. Boxplots show medians and upper and lower quartiles. The whiskers show 5 to 95 percentiles. Each dot represents an individual sample. * $P < 0.05$.

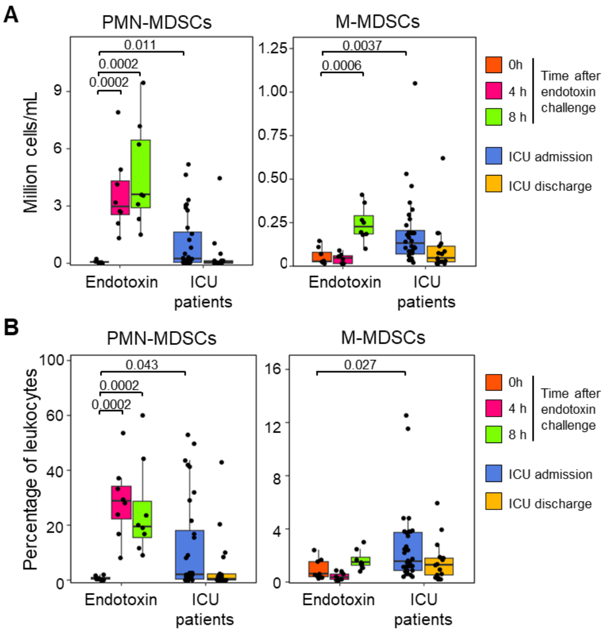

Figure S4. Comparison of MDSC-like cells levels between healthy subjects challenged with endotoxin and ICU patients. Absolute counts (**A**) and percentages (**B**) of PMN-MDSCs and M-MDSCs in healthy subjects (n = 8) infused for 0, 4 and 8 hours with endotoxin, and in ICU patients at study inclusion (n = 32) and at ICU discharge (n = 17). Boxplots show medians and upper and lower quartiles. The whiskers show 5 to 95 percentiles. *, P < 0.05; **, P ≤ 0.01; ***, P ≤ 0.001 (healthy patients before endotoxin challenge versus other groups of subjects).

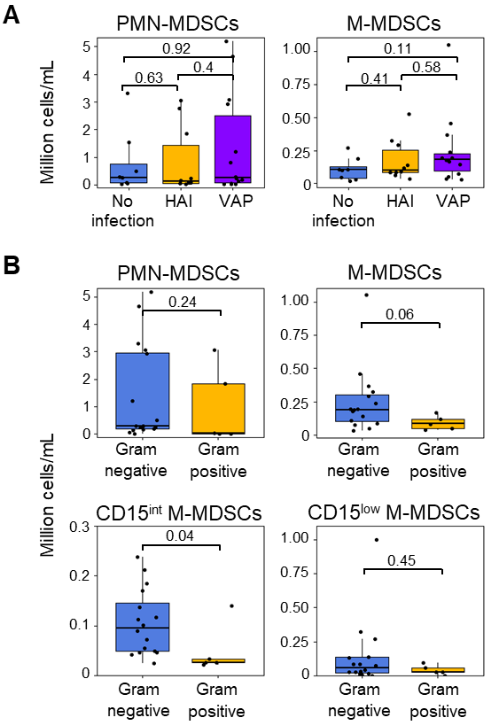

Figure S5. Absolute counts of MDSC-like cells in relation with the development of infection in mechanically ventilated ICU patients. **A**) PMN-MDSCs and M-MDSCs in patients who did not develop an infection (No HAI, n = 8), and in patients who developed hospital-acquired infection (HAI, other than ventilator-associated pneumonia-VAP, n = 10) and VAP (n = 14). **B**) PMN-MDSCs, M-MDSCs, CD15^{int} M-MDSCs and CD15^{low} M-MDSCs at ICU admission in patients who subsequently developed Gram-negative or Gram-positive bacterial infections. Boxplots show medians and upper and lower quartiles. The whiskers show 5 to 95 percentiles. Each dot represents an individual sample.

Table S1

Healthy volunteers challenged with with 2 ng/kg of LPS
Cell counts in blood

Healthy volunteer #1

Hours post endotoxin challenge	Leukocytes	Neutrophils	Neutrophils	PMN-MDSCs	PMN-MDSCs	Classical monocytes	Classical monocytes	Intermediate & non-classical monocytes	Intermediate & non-classical monocytes	M-MDSCs	M-MDSCs
	Million cells/mL	Million cells/mL	% of leukocytes	Million cells/mL	% of leukocytes	Million cells/mL	% of leukocytes	Million cells/mL	% of leukocytes	Million cells/mL	% of leukocytes
0	4.80	2.19	45.59	0.02	0.31	0.28	5.92	0.07	1.36	0.03	0.62
1	1.70	0.41	23.89	0.01	0.39	0.00	0.21	0.04	2.65	0.00	0.02
2	3.90	2.45	62.91	0.48	12.43	0.00	0.00	0.01	0.35	0.00	0.00
3	5.50	4.00	72.72	0.88	16.03	0.00	0.01	0.02	0.30	0.00	0.03
4	9.00	5.16	57.37	3.16	35.15	0.03	0.30	0.03	0.34	0.05	0.58
6	10.90	7.71	70.74	2.31	21.23	0.18	1.64	0.04	0.40	0.19	1.70
8	10.20	7.64	74.92	1.48	14.49	0.18	1.76	0.04	0.42	0.25	2.45
24	x	x	x	x	x	x	x	x	x	x	x
168	5.90	2.63	44.57	0.02	0.25	0.31	5.23	0.08	1.28	0.01	0.24

Healthy volunteer #2

Hours post endotoxin challenge	Leukocytes	Neutrophils	Neutrophils	PMN-MDSCs	PMN-MDSCs	Classical monocytes	Classical monocytes	Intermediate & non-classical monocytes	Intermediate & non-classical monocytes	M-MDSCs	M-MDSCs
	Million cells/mL	Million cells/mL	% of leukocytes	Million cells/mL	% of leukocytes	Million cells/mL	% of leukocytes	Million cells/mL	% of leukocytes	Million cells/mL	% of leukocytes
0	4.50	2.06	45.88	0.02	0.49	0.13	2.95	0.05	1.21	0.07	1.53
1	2.00	0.47	23.37	0.01	0.57	0.00	0.00	0.03	1.47	0.00	0.00
2	4.00	1.56	38.91	1.37	34.37	0.00	0.00	0.01	0.25	0.00	0.00
3	3.90	1.89	48.56	1.47	37.73	0.00	0.00	0.01	0.29	0.00	0.00
4	6.60	3.44	52.06	2.75	41.68	0.00	0.02	0.02	0.28	0.01	0.15
6	10.30	5.57	54.08	4.15	40.31	0.04	0.37	0.03	0.34	0.12	1.17
8	13.00	4.90	37.66	7.20	55.40	0.05	0.39	0.04	0.30	0.18	1.38
24	9.80	4.67	47.60	0.28	2.85	0.50	5.11	0.10	1.06	0.08	0.78
168	6.30	4.05	64.29	0.38	5.96	0.14	2.23	0.04	0.71	0.07	1.05

Healthy volunteer #3

Hours post endotoxin challenge	Leukocytes	Neutrophils	Neutrophils	PMN-MDSCs	PMN-MDSCs	Classical monocytes	Classical monocytes	Intermediate & non-classical monocytes	Intermediate & non-classical monocytes	M-MDSCs	M-MDSCs
	Million cells/mL	Million cells/mL	% of leukocytes	Million cells/mL	% of leukocytes	Million cells/mL	% of leukocytes	Million cells/mL	% of leukocytes	Million cells/mL	% of leukocytes
0	7.50	4.24	56.53	0.04	0.51	0.51	6.82	0.06	0.75	0.02	0.28
1	3.20	1.41	44.04	0.03	0.91	0.00	0.00	0.04	1.18	0.00	0.00
2	5.10	3.57	70.05	0.46	9.02	0.00	0.03	0.02	0.33	0.01	0.13
3	8.40	6.74	80.24	0.98	11.66	0.00	0.03	0.02	0.21	0.01	0.10
4	13.00	11.08	85.24	1.31	10.09	0.02	0.14	0.03	0.24	0.02	0.13
6	15.80	12.85	81.30	2.19	13.84	0.16	1.01	0.04	0.25	0.14	0.89
8	20.30	16.61	81.82	2.32	11.41	0.34	1.66	0.05	0.23	0.27	1.31
24	12.20	6.83	55.98	0.10	0.80	0.71	5.79	0.10	0.85	0.06	0.48
168	7.00	4.87	69.59	0.03	0.36	0.23	3.26	0.04	0.51	0.04	0.57

Healthy volunteer #4

Hours post endotoxin challenge	Leukocytes	Neutrophils	Neutrophils	PMN-MDSCs	PMN-MDSCs	Classical monocytes	Classical monocytes	Intermediate & non-classical monocytes	Intermediate & non-classical monocytes	M-MDSCs	M-MDSCs
	Million cells/mL	Million cells/mL	% of leukocytes	Million cells/mL	% of leukocytes	Million cells/mL	% of leukocytes	Million cells/mL	% of leukocytes	Million cells/mL	% of leukocytes
0	8.80	5.79	65.79	0.23	2.67	0.33	3.71	0.02	0.19	0.15	1.65
1	3.00	1.76	58.50	0.05	1.79	0.00	0.00	0.01	0.24	0.00	0.00
2	7.30	3.99	54.71	2.44	33.47	0.00	0.00	0.01	0.10	0.00	0.04
3	12.70	7.91	62.30	4.44	34.93	0.00	0.02	0.01	0.05	0.01	0.11
4	17.10	8.77	51.28	7.93	46.40	0.04	0.21	0.01	0.06	0.06	0.37
6	23.80	16.36	68.74	6.72	28.23	0.21	0.89	0.01	0.05	0.21	0.89
8	24.80	17.41	70.21	6.22	25.08	0.32	1.30	0.01	0.05	0.41	1.64
24	13.60	9.21	67.69	0.29	2.15	0.57	4.19	0.02	0.18	0.40	2.91
168	12.70	8.89	69.96	0.38	3.00	0.42	3.34	0.02	0.17	0.08	0.62

Healthy volunteer #5

Hours post endotoxin challenge	Leukocytes	Neutrophils	Neutrophils	PMN-MDSCs	PMN-MDSCs	Classical monocytes	Classical monocytes	Intermediate & non-classical monocytes	Intermediate & non-classical monocytes	M-MDSCs	M-MDSCs
	Million cells/mL	Million cells/mL	% of leukocytes	Million cells/mL	% of leukocytes	Million cells/mL	% of leukocytes	Million cells/mL	% of leukocytes	Million cells/mL	% of leukocytes
0	5.00	2.73	54.67	0.05	1.06	0.29	5.75	0.05	1.03	0.02	0.44
1	2.00	0.86	43.02	0.12	5.86	0.00	0.00	0.03	1.67	0.00	0.00
2	3.70	1.04	28.15	1.91	51.58	0.00	0.00	0.01	0.32	0.00	0.00
3	4.90	2.17	44.31	2.29	46.81	0.00	0.04	0.01	0.30	0.00	0.07
4	7.30	1.97	26.95	4.90	67.14	0.02	0.29	0.02	0.29	0.02	0.21
6	10.40	6.22	59.82	3.55	34.15	0.14	1.37	0.04	0.35	0.08	0.75
8	12.60	2.01	15.96	9.49	75.28	0.24	1.89	0.05	0.42	0.10	0.79
24	8.10	3.91	48.22	0.10	1.20	0.52	6.36	0.11	1.36	0.06	0.78
168	5.70	3.76	65.98	0.17	3.03	0.22	3.79	0.05	0.93	0.00	0.09

Healthy volunteer #6

Hours post endotoxin challenge	Leukocytes	Neutrophils	Neutrophils	PMN-MDSCs	PMN-MDSCs	Classical monocytes	Classical monocytes	Intermediate & non-classical monocytes	Intermediate & non-classical monocytes	M-MDSCs	M-MDSCs
	Million cells/mL	Million cells/mL	% of leukocytes	Million cells/mL	% of leukocytes	Million cells/mL	% of leukocytes	Million cells/mL	% of leukocytes	Million cells/mL	% of leukocytes
0	4.10	2.14	52.12	0.03	0.63	0.18	4.50	0.06	1.39	0.02	0.55
1	1.60	0.61	38.20	0.01	0.67	0.00	0.01	0.04	2.39	0.00	0.00
2	3.90	2.28	58.34	0.83	21.39	0.00	0.04	0.02	0.44	0.01	0.25
3	6.30	3.70	58.80	2.11	33.46	0.01	0.13	0.02	0.36	0.02	0.34
4	11.30	6.55	57.99	4.14	36.63	0.08	0.67	0.03	0.29	0.09	0.78
6	12.10	7.53	62.23	3.78	31.21	0.24	1.99	0.04	0.30	0.18	1.49
8	13.00	8.77	67.44	3.09	23.79	0.38	2.93	0.04	0.31	0.20	1.50
24	7.80	4.25	54.51	0.03	0.41	0.36	4.63	0.10	1.32	0.03	0.43
168	5.20	3.55	68.30	0.15	2.89	0.20	3.87	0.05	0.95	0.01	0.17

Healthy volunteer #7

Hours post endotoxin challenge	Leukocytes	Neutrophils	Neutrophils	PMN-MDSCs	PMN-MDSCs	Classical monocytes	Classical monocytes	Intermediate & non-classical monocytes	Intermediate & non-classical monocytes	M-MDSCs	M-MDSCs
	Million cells/mL	Million cells/mL	% of leukocytes	Million cells/mL	% of leukocytes	Million cells/mL	% of leukocytes	Million cells/mL	% of leukocytes	Million cells/mL	% of leukocytes
0	4.40	2.51	57.01	0.01	0.23	0.28	6.36	0.02	0.37	0.02	0.37
1	2.20	1.17	53.34	0.01	0.45	0.00	0.00	0.01	0.50	0.00	0.00
2	5.20	3.49	67.16	0.75	14.37	0.00	0.05	0.01	0.14	0.01	0.16
3	7.50	5.64	75.24	1.40	18.61	0.00	0.06	0.01	0.10	0.01	0.11
4	12.60	9.46	75.09	2.67	21.17	0.03	0.22	0.01	0.06	0.04	0.32
6	16.80	13.19	78.51	2.73	16.28	0.26	1.53	0.01	0.05	0.18	1.06
8	16.70	11.63	69.67	3.55	21.24	0.51	3.05	0.01	0.07	0.18	1.09
24	8.90	4.90	55.04	0.04	0.44	0.31	3.44	0.03	0.30	0.01	0.15
168	6.20	3.88	62.61	0.07	1.21	0.25	4.01	0.02	0.30	0.01	0.20

Healthy volunteer #8

Hours post endotoxin challenge	Leukocytes	Neutrophils	Neutrophils	PMN-MDSCs	PMN-MDSCs	Classical monocytes	Classical monocytes	Intermediate & non-classical monocytes	Intermediate & non-classical monocytes	M-MDSCs	M-MDSCs
	Million cells/mL	Million cells/mL	% of leukocytes	Million cells/mL	% of leukocytes	Million cells/mL	% of leukocytes	Million cells/mL	% of leukocytes	Million cells/mL	% of leukocytes
0	4.50	2.38	52.98	0.11	2.34	0.20	4.40	0.07	1.55	0.11	2.39
1	2.00	0.72	36.23	0.02	0.81	0.00	0.00	0.05	2.65	0.00	0.00
2	2.60	1.27	48.83	0.54	20.90	0.00	0.00	0.02	0.68	0.00	0.03
3	3.50	2.11	60.18	1.08	30.87	0.00	0.04	0.02	0.52	0.02	0.45
4	7.00	4.51	64.46	2.11	30.08	0.01	0.12	0.04	0.52	0.06	0.86
6	9.80	4.99	50.92	4.29	43.78	0.06	0.58	0.05	0.49	0.12	1.24
8	12.20	7.42	60.80	3.61	29.60	0.13	1.10	0.06	0.50	0.36	2.98
24	7.40	3.03	40.98	0.04	0.59	0.21	2.83	0.10	1.33	0.27	3.71

**168**	4.20	2.46	58.60	0.14	3.36	0.18	4.23	0.05	1.14	0.03	0.80

x : not available

Mean of 8 healthy volunteers

Hours post endotoxin infusion	Leukocytes Million cells/mL	Neutrophils Million cells/mL	Neutrophils % of leukocytes	PMN-MDSCs Million cells/mL	PMN-MDSCs % of leukocytes	Classical monocytes Million cells/mL	Classical monocytes % of leukocytes	Intermediate & non-classical monocytes Million cells/mL	Intermediate & non-classical monocytes % of leukocytes	M-MDSCs Million cells/mL	M-MDSCs % of leukocytes
**0**	5.45	3.01	53.82	0.06	1.03	0.28	5.05	0.05	0.98	0.05	0.98
**1**	2.21	0.93	40.07	0.03	1.43	0.00	0.03	0.03	1.59	0.00	0.00
**2**	4.46	2.46	53.63	1.10	24.69	0.00	0.02	0.01	0.33	0.00	0.08
**3**	6.59	4.27	62.80	1.83	28.76	0.00	0.04	0.01	0.27	0.01	0.15
**4**	10.49	6.37	58.81	3.62	36.04	0.03	0.25	0.02	0.26	0.04	0.43
**6**	13.74	9.30	65.79	3.72	28.63	0.16	1.17	0.03	0.28	0.15	1.15
**8**	15.35	9.55	59.81	4.62	32.04	0.27	1.76	0.04	0.29	0.24	1.64
**24**	9.69	5.26	52.86	0.13	1.21	0.45	4.62	0.08	0.92	0.13	1.32
**168**	6.65	4.26	62.99	0.17	2.51	0.24	3.75	0.04	0.75	0.03	0.47

Median of 8 healthy volunteers

Hours post endotoxin infusion	Leukocytes Million cells/mL	Neutrophils Million cells/mL	Neutrophils % of leukocytes	PMN-MDSCs Million cells/mL	PMN-MDSCs % of leukocytes	Classical monocytes Million cells/mL	Classical monocytes % of leukocytes	Intermediate & non-classical monocytes Million cells/mL	Intermediate & non-classical monocytes % of leukocytes	M-MDSCs Million cells/mL	M-MDSCs % of leukocytes
**0**	4.65	2.45	53.82	0.03	0.57	0.28	5.12	0.06	1.12	0.03	0.58
**1**	2.00	0.79	40.61	0.01	0.74	0.00	0.00	0.04	1.57	0.00	0.00
**2**	3.95	2.36	56.52	0.79	21.14	0.00	0.00	0.01	0.33	0.00	0.04
**3**	5.90	3.85	61.24	1.43	32.17	0.00	0.03	0.02	0.29	0.01	0.10
**4**	10.15	5.86	57.68	2.96	35.89	0.02	0.22	0.03	0.28	0.05	0.35
**6**	11.50	7.62	65.49	3.66	29.72	0.17	1.19	0.04	0.32	0.16	1.11
**8**	13.00	8.20	68.55	3.58	24.43	0.28	1.71	0.04	0.31	0.22	1.44
**24**	8.90	4.67	54.51	0.10	0.80	0.50	4.63	0.10	1.06	0.06	0.78
**168**	6.05	3.82	65.14	0.15	2.94	0.22	3.83	0.05	0.82	0.02	0.40

SD of 8 healthy volunteers

Hours post endotoxin infusion	Leukocytes Million cells/mL	Neutrophils Million cells/mL	Neutrophils % of leukocytes	PMN-MDSCs Million cells/mL	PMN-MDSCs % of leukocytes	Classical monocytes Million cells/mL	Classical monocytes % of leukocytes	Intermediate & non-classical monocytes Million cells/mL	Intermediate & non-classical monocytes % of leukocytes	M-MDSCs Million cells/mL	M-MDSCs % of leukocytes
**0**	1.72	1.33	6.50	0.08	0.95	0.12	1.36	0.02	0.50	0.05	0.77
**1**	0.58	0.48	12.53	0.04	1.84	0.00	0.07	0.02	0.93	0.00	0.01
**2**	1.41	1.13	14.40	0.74	14.25	0.00	0.02	0.00	0.18	0.00	0.09
**3**	2.98	2.28	12.68	1.17	12.12	0.00	0.04	0.01	0.15	0.01	0.16
**4**	3.68	3.17	17.38	2.07	17.06	0.02	0.19	0.01	0.15	0.03	0.29
**6**	4.84	4.23	10.97	1.46	10.89	0.08	0.56	0.01	0.16	0.04	0.32
**8**	4.94	5.39	22.01	2.74	22.00	0.15	0.89	0.02	0.16	0.10	0.73
**24**	4.06	2.69	20.26	0.11	0.97	0.22	2.00	0.05	0.56	0.14	1.37
**168**	2.58	2.02	8.36	0.14	1.88	0.09	0.86	0.02	0.40	0.03	0.35

Healthy volunteers challenged with with 2 ng/kg of LPS
Cell counts in blood

Healthy volunteer #	Hours post endotoxin challenge	Leukocytes	Neutrophils	Neutrophils	PMN-MDSCs	PMN-MDSCs	Classical monocytes	Classical monocytes	Intermediate & non-classical monocytes	Intermediate & non-classical monocytes	Total monocytes	M-MDSCs	M-MDSCs	Total monocytic cells	Classical monocytes	Intermediate & non-classical monocytes	M-MDSCs	
		Million cells/mL	Million cells/mL	% of leukocytes	Million cells/mL	% of leukocytes	Million cells/mL	% of leukocytes	Million cells/mL	% of leukocytes	Million cells/mL	Million cells/mL	% of leukocytes	Million cells/mL	% of monocytic cells	% of monocytic cells	% of monocytic cells	Total %
1	0	4.800	2.188	45.593	0.015	0.313	0.284	5.924	0.065	1.357	0.350	0.030	0.621	0.379	74.96	17.18	7.86	100.00
2	0	4.500	2.064	45.877	0.022	0.487	0.133	2.950	0.055	1.213	0.187	0.069	1.534	0.256	51.79	21.29	26.92	100.00
3	0	7.500	4.240	56.533	0.038	0.506	0.512	6.824	0.056	0.748	0.568	0.021	0.284	0.589	86.87	9.52	3.61	100.00
4	0	8.800	5.790	65.794	0.235	2.669	0.327	3.713	0.017	0.193	0.344	0.145	1.648	0.489	66.86	3.47	29.67	100.00
5	0	5.000	2.734	54.671	0.053	1.062	0.287	5.745	0.051	1.029	0.339	0.022	0.443	0.361	79.60	14.26	6.14	100.00
6	0	4.100	2.137	52.120	0.026	0.631	0.184	4.495	0.057	1.395	0.241	0.022	0.547	0.264	69.84	21.67	8.50	100.00
7	0	4.400	2.509	57.013	0.010	0.227	0.280	6.356	0.016	0.370	0.296	0.016	0.370	0.312	89.57	5.22	5.21	100.00
8	0	4.500	2.384	52.977	0.105	2.336	0.198	4.397	0.070	1.546	0.267	0.107	2.386	0.375	52.79	18.56	28.65	100.00
Median		4.650	2.446	53.824	0.032	0.569	0.282	5.120	0.055	1.121	0.317	0.026	0.584	0.368	72.400	15.718	8.177	
Mean		5.450	3.006	53.822	0.063	1.029	0.276	5.051	0.048	0.981	0.324	0.054	0.979	0.378	71.534	13.895	14.571	100.00
SD		1.723	1.325	6.503	0.076	0.947	0.116	1.365	0.020	0.498	0.114	0.048	0.774	0.113	14.157	7.080	11.585	
Q1 IQR		4.475	2.176	50.559	0.020	0.443	0.194	4.226	0.043	0.654	0.261	0.022	0.425	0.300	63.343	8.445	5.911	
Q3 IQR		5.625	3.110	56.653	0.066	1.381	0.297	6.032	0.059	1.367	0.345	0.079	1.562	0.407	81.416	19.242	27.355	
1	1	1.70	0.41	23.89	0.01	0.39	0.00	0.21	0.04	2.65	0.049	0.00	0.02	0.049	7.27	92.11	0.63	100.00
2	1	2.00	0.47	23.37	0.01	0.57	0.00	0.00	0.03	1.47	0.029	0.00	0.00	0.029	0.00	100.00	0.00	100.00
3	1	3.20	1.41	44.04	0.03	0.91	0.00	0.00	0.04	1.18	0.038	0.00	0.00	0.038	0.04	99.96	0.00	100.00
4	1	3.00	1.76	58.50	0.05	1.79	0.00	0.00	0.01	0.24	0.007	0.00	0.00	0.007	0.60	99.40	0.00	100.00
5	1	2.00	0.86	43.02	0.12	5.86	0.00	0.00	0.03	1.67	0.033	0.00	0.00	0.033	0.20	99.80	0.00	100.00
6	1	1.60	0.61	38.20	0.01	0.67	0.00	0.01	0.04	2.39	0.038	0.00	0.00	0.038	0.33	99.63	0.04	100.00
7	1	2.20	1.17	53.34	0.01	0.45	0.00	0.00	0.01	0.50	0.011	0.00	0.00	0.011	0.13	99.87	0.00	100.00
8	1	2.00	0.72	36.23	0.02	0.81	0.00	0.00	0.05	2.65	0.053	0.00	0.00	0.053	0.00	100.00	0.00	100.00
Median		2.000	0.793	40.609	0.014	0.737	0.000	0.001	0.036	1.568	0.036	0.000	0.000	0.036	0.166	99.834	0.000	
Mean		2.213	0.926	40.074	0.032	1.430	0.000	0.028	0.032	1.592	0.032	0.000	0.002	0.032	1.072	98.845	0.083	100.00
SD		0.582	0.479	12.533	0.038	1.845	0.001	0.073	0.016	0.931	0.016	0.000	0.006	0.016	2.512	2.731	0.220	
Q1 IQR		1.925	0.575	33.146	0.010	0.536	0.000	0.000	0.025	1.012	0.025	0.000	0.000	0.025	0.032	99.573	0.000	
Q3 IQR		2.400	1.232	46.369	0.035	1.130	0.000	0.005	0.040	2.451	0.041	0.000	0.000	0.041	0.399	99.968	0.009	
1	2	3.90	2.45	62.91	0.48	12.43	0.00	0.00	0.01	0.35	0.014	0.00	0.00	0.014	0.00	100.00	0.00	100.00
2	2	4.00	1.56	38.91	1.37	34.37	0.00	0.00	0.01	0.25	0.010	0.00	0.00	0.010	0.34	99.16	0.50	100.00
3	2	5.10	3.57	70.05	0.46	9.02	0.00	0.03	0.02	0.33	0.019	0.01	0.13	0.025	6.73	67.07	26.20	100.00
4	2	7.30	3.99	54.71	2.44	33.47	0.00	0.00	0.01	0.10	0.007	0.00	0.04	0.010	3.04	68.21	28.75	100.00
5	2	3.70	1.04	28.15	1.91	51.58	0.00	0.00	0.01	0.32	0.012	0.00	0.00	0.012	0.00	100.00	0.00	100.00
6	2	3.90	2.28	58.34	0.83	21.39	0.00	0.04	0.02	0.44	0.019	0.01	0.25	0.028	5.68	60.30	34.02	100.00
7	2	5.20	3.49	67.16	0.75	14.37	0.00	0.05	0.01	0.14	0.010	0.01	0.16	0.018	15.44	39.54	45.03	100.00
8	2	2.60	1.27	48.83	0.54	20.90	0.00	0.00	0.02	0.68	0.018	0.00	0.03	0.019	0.36	95.14	4.50	100.00
Median		3.950	2.364	56.524	0.791	21.142	0.000	0.003	0.013	0.328	0.013	0.002	0.036	0.016	1.697	81.677	15.349	
Mean		4.463	2.457	53.633	1.099	24.689	0.001	0.017	0.013	0.326	0.013	0.004	0.076	0.017	3.947	78.678	17.375	100.00
SD		1.409	1.130	14.397	0.739	14.250	0.001	0.022	0.004	0.182	0.004	0.004	0.092	0.007	5.354	23.034	18.139	
Q1 IQR		3.850	1.485	46.352	0.529	13.886	0.000	0.001	0.009	0.221	0.010	0.000	0.001	0.012	0.252	65.377	0.378	
Q3 IQR		5.125	3.512	63.972	1.508	33.694	0.002	0.035	0.017	0.372	0.018	0.007	0.137	0.020	5.941	99.370	30.070	
1	3	5.50	4.00	72.72	0.88	16.03	0.00	0.01	0.02	0.30	0.017	0.00	0.03	0.018	2.68	88.77	8.55	100.00
2	3	3.90	1.89	48.56	1.47	37.73	0.00	0.00	0.01	0.29	0.011	0.00	0.00	0.011	0.13	99.12	0.76	100.00
3	3	8.40	6.74	80.24	0.98	11.66	0.00	0.03	0.02	0.21	0.020	0.01	0.10	0.028	8.48	63.41	28.11	100.00
4	3	12.70	7.91	62.30	4.44	34.93	0.00	0.02	0.01	0.05	0.008	0.01	0.11	0.022	10.10	28.39	61.51	100.00
5	3	4.90	2.17	44.31	2.29	46.81	0.00	0.04	0.01	0.30	0.017	0.00	0.07	0.020	8.93	74.84	16.23	100.00
6	3	6.30	3.70	58.80	2.11	33.46	0.01	0.13	0.02	0.36	0.031	0.02	0.34	0.052	15.43	43.58	40.99	100.00
7	3	7.50	5.64	75.24	1.40	18.61	0.00	0.06	0.01	0.10	0.012	0.01	0.11	0.020	22.01	37.32	40.67	100.00
8	3	3.50	2.11	60.18	1.08	30.87	0.00	0.04	0.02	0.52	0.019	0.02	0.45	0.035	3.79	51.59	44.61	100.00
Median		5.900	3.852	61.242	1.434	32.167	0.002	0.032	0.016	0.293	0.017	0.008	0.101	0.021	8.709	57.499	34.391	
Mean		6.588	4.271	62.795	1.831	28.765	0.003	0.039	0.014	0.266	0.017	0.009	0.149	0.026	8.944	60.877	30.179	100.00
SD		2.984	2.280	12.679	1.170	12.120	0.003	0.040	0.006	0.148	0.007	0.007	0.158	0.013	7.150	25.178	20.527	
Q1 IQR		4.650	2.155	56.242	1.055	17.969	0.001	0.015	0.010	0.186	0.012	0.003	0.056	0.019	3.514	42.014	14.309	
Q3 IQR		7.725	5.917	73.353	2.155	35.632	0.003	0.043	0.018	0.316	0.020	0.014	0.165	0.030	11.431	78.320	41.899	
1	4	9.00	5.16	57.37	3.16	35.15	0.03	0.30	0.03	0.34	0.057	0.05	0.58	0.110	24.19	27.97	47.83	100.00
2	4	6.60	3.44	52.06	2.75	41.68	0.00	0.02	0.02	0.28	0.020	0.01	0.15	0.030	3.41	63.17	33.42	100.00
3	4	13.00	11.08	85.24	1.31	10.09	0.02	0.14	0.03	0.24	0.050	0.02	0.13	0.066	28.02	47.35	24.63	100.00
4	4	17.10	8.77	51.28	7.93	46.40	0.04	0.21	0.01	0.06	0.046	0.06	0.37	0.109	32.84	9.01	58.15	100.00
5	4	7.30	1.97	26.95	4.90	67.14	0.02	0.29	0.02	0.29	0.042	0.02	0.21	0.058	36.74	36.74	26.52	100.00
6	4	11.30	6.55	57.99	4.14	36.63	0.08	0.67	0.03	0.29	0.108	0.09	0.78	0.196	38.47	16.40	45.13	100.00
7	4	12.60	9.46	75.09	2.67	21.17	0.03	0.22	0.01	0.06	0.036	0.04	0.32	0.076	36.79	9.91	53.29	100.00
8	4	7.00	4.51	64.46	2.11	30.08	0.01	0.12	0.04	0.52	0.045	0.06	0.86	0.106	8.14	34.58	57.28	100.00
Median		10.150	5.858	57.680	2.957	35.888	0.024	0.217	0.026	0.285	0.045	0.047	0.348	0.091	30.430	31.275	46.480	
Mean		10.488	6.368	58.807	3.622	36.041	0.027	0.246	0.024	0.260	0.050	0.043	0.427	0.094	26.076	30.642	43.282	100.00
SD		3.680	3.172	17.383	2.071	17.060	0.023	0.194	0.011	0.150	0.026	0.028	0.286	0.050	13.473	18.889	13.455	
Q1 IQR		7.225	4.243	51.869	2.527	27.853	0.016	0.138	0.017	0.196	0.041	0.016	0.195	0.064	20.182	14.782	31.695	
Q3 IQR		12.700	8.943	67.120	4.329	42.856	0.030	0.292	0.032	0.303	0.052	0.061	0.634	0.109	36.752	39.391	54.289	
1	6	10.90	7.71	70.74	2.31	21.23	0.18	1.64	0.04	0.40	0.223	0.19	1.70	0.409	43.83	10.76	45.40	100.00
2	6	10.30	5.57	54.08	4.15	40.31	0.04	0.37	0.03	0.34	0.073	0.12	1.17	0.193	19.55	18.01	62.45	100.00
3	6	15.80	12.85	81.30	2.19	13.84	0.16	1.01	0.04	0.25	0.198	0.14	0.89	0.339	46.95	11.48	41.57	100.00
4	6	23.80	16.36	68.74	6.72	28.23	0.21	0.89	0.01	0.05	0.225	0.21	0.89	0.437	48.66	2.74	48.60	100.00
5	6	10.40	6.22	59.82	3.55	34.15	0.14	1.37	0.04	0.35	0.179	0.08	0.75	0.258	55.19	14.33	30.48	100.00
6	6	12.10	7.53	62.23	3.78	31.21	0.24	1.99	0.04	0.30	0.277	0.18	1.49	0.457	52.63	7.91	39.46	100.00
7	6	16.80	13.19	78.51	2.73	16.28	0.26	1.53	0.01	0.05	0.267	0.18	1.06	0.445	58.01	2.05	39.95	100.00
8	6	9.80	4.99	50.92	4.29	43.78	0.06	0.58	0.05	0.49	0.105	0.12	1.24	0.226	25.18	21.26	53.55	100.00
Median		11.500	7.621	65.486	3.664	29.719	0.169	1.187	0.037	0.318	0.211	0.159	1.114	0.374	47.807	11.123	43.485	
Mean		13.738	9.302	65.792	3.715	28.628	0.161	1.173	0.032	0.280	0.193	0.152	1.150	0.345	43.751	11.068	45.181	100.00
SD		4.841	4.229	10.968	1.456	10.885	0.080	0.556	0.014	0.158	0.073	0.044	0.322	0.107	14.031	6.801	9.778	
Q1 IQR		10.375	6.058	58.382	2.630	19.996	0.121	0.816	0.029	0.198	0.161	0.121	0.893	0.250	39.172	6.615	39.824	
Q3 IQR		16.050	12.931	72.684	4.186	35.688	0.220	1.562	0.040	0.367	0.235	0.182	1.300	0.439	53.273	15.249	49.835	
1	8	10.20	7.64	74.92	1.48	14.49	0.18	1.76	0.04	0.42	0.222	0.25	2.45	0.472	38.06	9.00	52.94	100.00
2	8	13.00	4.90	37.66	7.20	55.40	0.05	0.39	0.04	0.30	0.090	0.18	1.38	0.270	18.89	14.62	66.49	100.00
3	8	20.30	16.61	81.82	2.32	11.41	0.34	1.66	0.05	0.23	0.385	0.27	1.31	0.651	51.80	7.30	40.90	100.00
4	8	24.80	17.41	70.21	6.22	25.08	0.32	1.30	0.01	0.05	0.334	0.41	1.64	0.742	43.33	1.73	54.93	100.00
5	8	12.60	2.01	15.96	9.49	75.28	0.24	1.89	0.05	0.42	0.291	0.10	0.79	0.390	60.92	13.54	25.54	100.00
6	8	13.00	8.77	67.44	3.09	23.79	0.38	2.93	0.04	0.31	0.422	0.20	1.50	0.618	61.75	6.59	31.66	100.00
7	8	16.70	11.63	69.67	3.55	21.24	0.51	3.05	0.01	0.07	0.522	0.18	1.09	0.705	72.34	1.76	25.90	100.00
8	8	12.20	7.42	60.80	3.61	29.60	0.13	1.10	0.06	0.50	0.195	0.36	2.98	0.559	24.00	10.86	65.13	100.00
Median		13.000	8.204	68.551	3.579	24.434	0.280	1.712	0.042	0.308	0.312	0.223	1.442	0.588	47.567	8.149	46.923	
Mean		15.350	9.549	59.809	4.619	32.035	0.269	1.761	0.039	0.289	0.308	0.243	1.645	0.551	46.387	8.176	45.438	100.00
SD		4.938	5.389	22.007	2.741	22.002	0.148	0.894	0.017	0.162	0.138	0.102	0.725	0.163	18.843	4.843	16.756	
Q1 IQR		12.500	6.787	55.013	2.898	19.552	0.168	1.247	0.033	0.194	0.215	0.182	1.257	0.452	34.543	5.383	30.224	
Q3 IQR		17.600	12.878	71.387	6.465	36.049	0.348	2.148	0.049	0.418	0.394	0.291	1.846	0.665	61.129	11.532	57.483	
1	24	x	x	x	x	x	x	x	x	x	x	x	x	x	x	x	x	0.00
2	24	9.80	4.67	47.60	0.28	2.85	0.50	5.11	0.10	1.06	0.605	0.08	0.78	0.681	73.51	15.32	11.17	100.00
3	24	12.20	6.83	55.98	0.10	0.80	0.71	5.79	0.10	0.85	0.810	0.06	0.48	0.869	81.26	11.97	6.77	100.00
4	24	13.60	9.21	67.69	0.29	2.15	0.57	4.19	0.02	0.18	0.593	0.40	2.91	0.990	57.52	2.43	40.05	100.00
5	24	8.10	3.91	48.22	0.10	1.20	0.52	6.36	0.11	1.36	0.626	0.06	0.78	0.689	74.78	16.01	9.22	100.00
6	24	7.80	4.25	54.51	0.03	0.41	0.36	4.63	0.10	1.32	0.464	0.03	0.43	0.498	72.49	20.71	6.80	100.00
7	24	8.90	4.90	55.04	0.04	0.44	0.31	3.44	0.03	0.30	0.333	0.01	0.15	0.346	88.46	7.69	3.85	100.00
8	24	7.40	3.03	40.98	0.04	0.59	0.21	2.83	0.10	1.33	0.308	0.27	3.71	0.582	35.98	16.92	47.11	100.00
Median		8.900	4.665	54.507	0.097	0.800	0.501	4.630	0.103	1.065	0.593	0.064	0.777	0.681	73.509	15.316	9.216	
Mean		9.686	5.256	52.860	0.126	1.206	0.453	4.621	0.082	0.916	0.534	0.131	1.321	0.665	69.141	13.006	17.853	100.00
SD		4.065	2.686	20.256	0.113	0.972	0.224	2.004	0.046	0.564	0.251	0.142	1.373	0.310	29.277	7.352	17.678	
Q1 IQR		7.950	4.079	47.913	0.041	0.515	0.334	3.813	0.063	0.576	0.399	0.046	0.458	0.540	65.006	9.831	6.784	
Q3 IQR		11.000	5.864	55.508	0.188	1.675	0.542	5.450	0.104	1.327	0.615	0.175	1.849	0.779	78.018	16.463	25.613	
1	168	5.90	2.63	44.57	0.02	0.25	0.31	5.23	0.08	1.28	0.384	0.01	0.24	0.398	77.53	18.92	3.55	100.00
2	168	6.30	4.05	64.29	0.38	5.96	0.14	2.23	0.04	0.71	0.185	0.07	1.05	0.251	55.80	17.79	26.42	100.00
3	168	7.00	4.87	69.59	0.03	0.36	0.23	3.26	0.04	0.51	0.264	0.04	0.57	0.304	75.21	11.66	13.14	100.00
4	168	12.70	8.89	69.96	0.38	3.00	0.42	3.34	0.02	0.17	0.445	0.08	0.62	0.524	80.85	4.03	15.12	100.00
5	168	5.70	3.76	65.98	0.17	3.03	0.22	3.79	0.05	0.93	0.269	0.00	0.09	0.274	78.84	19.38	1.78	100.00
6	168	5.20	3.55	68.30	0.15	2.89	0.20	3.87	0.05	0.95	0.251	0.01	0.17	0.260	77.57	19.08	3.35	100.00
7	168	6.20	3.88	62.61	0.07	1.21	0.25	4.01	0.02	0.30	0.268	0.01	0.20	0.280	88.85	6.72	4.43	100.00
8	168	4.20	2.46	58.60	0.14	3.36	0.18	4.23	0.05	1.14	0.226	0.03	0.80	0.259	68.60	18.49	12.90	100.00
Median		6.050	3.821	65.137	0.146	2.944	0.222	3.832	0.046	0.821	0.266	0.024	0.405	0.277	77.550	18.140	8.666	
Mean		6.650	4.262	62.989	0.167	2.508	0.243	3.745	0.043	0.748	0.286	0.032	0.467	0.319	75.405	14.509	10.086	100.00
SD		2.581	2.021	8.363	0.142	1.877	0.088	0.864	0.018	0.397	0.085	0.028	0.348	0.095	9.726	6.203	8.437	
Q1 IQR		5.575	3.321	61.609	0.063	0.997	0.195	3.318	0.032	0.455	0.245	0.011	0.192	0.259	73.556	10.423	3.497	
Q3 IQR		6.475	4.256	68.622	0.223	3.110	0.264	4.067	0.050	1.000	0.298	0.047	0.667	0.327	79.341	18.963	13.632	

x : not available

Healthy volunteers challenged with with 2 ng/kg of LPS
Cytokines in blood

CCL3, MIP1α (pg/ml)														
Healthy volunteer #		1	2	3	4	5	6	7	8	Median	Mean	SD	Q1 IQR	Q3 IQR
Hours post	0	50.0	32.9	29.0	8.7	15.2	20.0	6.6	14.4					
endotoxin challenge	0	52.2	27.1	17.0	4.8	13.6	18.9	5.1	10.0	16.1	20.3	14.6	9.7	27.6
	1	255.0	284.0	435.0	840.0	284.0	314.0	205.0	297.0	290.5	364.3	203.0	276.8	344.3
	2	556.0	501.0	647.0	704.0	409.0	274.0	248.0	348.0	455.0	460.9	169.1	329.5	578.8
	3	138.0	261.0	179.0	248.0	114.0	127.0	97.5	67.8	132.5	154.0	69.8	109.9	196.3
	4	82.6	86.6	136.0	187.0	72.9	64.4	46.0	45.6	77.8	90.1	48.5	59.8	98.9
	6	64.4	44.0	93.3	150.0	39.4	35.0	19.6	23.3	41.7	58.6	43.9	32.1	71.6
	8	62.6	39.2	48.0	91.6	23.8	27.4	10.7	24.3	33.3	41.0	26.1	24.2	51.7
	336	43.7	24.6	15.4	10.2	16.2	21.8	6.6	8.5	15.8	18.4	12.0	9.7	22.5

IL-6 (pg/ml)														
Healthy volunteer #		1	2	3	4	5	6	7	8	Median	Mean	SD	Q1 IQR	Q3 IQR
Hours post	0	35.0	3.2	43.8	3.2	29.4	10.5	23.0	12.1					
endotoxin challenge	0	41.2	3.2	11.4	3.2	25.8	8.7	19.3	6.2	11.8	17.5	14.0	5.5	26.7
	1	67.6	40.2	78.1	130.0	76.7	94.5	45.7	138.0	77.4	83.9	35.6	62.1	103.4
	2	1261.0	761.0	1758.0	1770.0	1048.0	824.0	863.0	1636.0	1154.5	1240.1	428.9	853.3	1666.5
	3	585.0	889.0	1578.0	1359.0	412.0	609.0	744.0	1228.0	816.5	925.5	417.3	603.0	1260.8
	4	118.0	590.0	718.0	378.0	177.0	133.0	285.0	340.0	312.5	342.4	216.6	166.0	431.0
	6	29.7	27.3	46.1	21.0	39.8	21.8	30.5	41.0	30.1	32.2	9.2	25.9	40.1
	8	35.6	4.5	22.4	6.3	34.2	12.2	21.6	18.5	20.1	19.4	11.6	10.7	25.4
	336	27.3	3.2	8.4	3.2	26.9	14.3	18.8	5.8	11.4	13.5	10.0	5.2	20.8

CCL4, MIP-1β (pg/ml)														
Healthy volunteer #		1	2	3	4	5	6	7	8	Median	Mean	SD	Q1 IQR	Q3 IQR
Hours post	0	31.0	31.0	31.0	31.1	31.1	31.1	31.1	31.1					
endotoxin challenge	0	63.8	103.0	97.5	64.2	60.7	71.7	28.9	66.7	31.1	50.3	25.3	31.0	64.8
	1	63.8	78.6	64.6	59.6	54.5	59.2	19.2	39.7	59.4	54.9	18.1	50.8	64.0
	2	5057.0	4134.0	6820.0	*10000.0*	5579.0	2611.0	2580.0	6154.0	5318.0	5366.9	2426.6	3753.3	6320.5
	3	2561.0	3114.0	3434.0	6958.0	2001.0	1394.0	1632.0	1507.0	2281.0	2825.1	1832.0	1600.8	3194.0
	4	732.0	956.0	1073.0	3822.0	851.0	415.0	622.0	372.0	791.5	1105.4	1124.8	570.3	985.3
	6	663.0	230.0	724.0	2049.0	510.0	150.0	253.0	193.0	381.5	596.5	627.1	220.8	678.3
	8	453.0	197.0	467.0	1615.0	224.0	126.0	151.0	241.0	232.5	434.3	494.1	185.5	456.5
	336	57.8	67.7	61.3	53.3	60.2	81.3	28.9	41.5	59.0	56.5	15.9	50.3	62.9

IL-8 (pg/ml)														
Healthy volunteer #		1	2	3	4	5	6	7	8	Median	Mean	SD	Q1 IQR	Q3 IQR
Hours post	0	20.0	15.0	12.0	6.0	11.0	10.0	9.0	14.0					
endotoxin challenge	0	25.0	13.0	5.0	3.0	12.0	8.0	8.0	9.0	10.5	11.3	5.5	8.0	13.3
	1	34.0	42.0	25.0	24.0	35.0	41.0	24.0	37.0	34.5	32.8	7.5	24.8	38.0
	2	784.0	1400.0	526.0	566.0	678.0	742.0	618.0	742.0	710.0	757.0	275.2	605.0	752.5
	3	567.0	1966.0	501.0	402.0	347.0	622.0	543.0	578.0	555.0	690.8	523.5	476.3	589.0
	4	186.0	548.0	175.0	184.0	187.0	204.0	219.0	227.0	195.5	241.3	125.3	185.5	221.0
	6	103.0	156.0	94.0	121.0	81.0	115.0	103.0	132.0	109.0	113.1	23.5	100.8	123.8
	8	100.0	115.0	64.0	85.0	56.0	77.0	57.0	110.0	81.0	83.0	23.4	62.3	102.5
	336	17.0	9.0	4.0	3.0	12.0	11.0	7.0	9.0	9.0	9.0	4.5	6.3	11.3

TNF (pg/ml)														
Healthy volunteer #		1	2	3	4	5	6	7	8	Median	Mean	SD	Q1 IQR	Q3 IQR
Hours post	0	26.0	38.0	48.0	26.0	21.0	35.0	11.0	29.0					
endotoxin challenge	0	19.0	29.0	39.0	25.0	24.0	32.0	10.0	13.0	26.0	26.6	10.5	20.5	32.8
	1	2351.0	4191.0	3911.0	4136.0	2671.0	3166.0	1280.0	1665.0	2918.5	2921.4	1120.4	2179.5	3967.3
	2	1868.0	4324.0	3771.0	3074.0	1849.0	1585.0	1171.0	1372.0	1858.5	2376.8	1186.2	1531.8	3248.3
	3	768.0	1445.0	1580.0	1111.0	561.0	786.0	518.0	408.0	777.0	897.1	437.1	550.3	1194.5
	4	400.0	836.0	747.0	611.0	432.0	416.0	322.0	199.0	424.0	495.4	217.1	380.5	645.0
	6	141.0	388.0	384.0	333.0	223.0	244.0	97.0	132.0	233.5	242.8	115.5	138.8	345.8
	8	124.0	271.0	311.0	224.0	141.0	168.0	54.0	105.0	154.5	174.8	87.4	119.3	235.8
	336	17.0	23.0	33.0	23.0	23.0	39.0	10.0	14.0	23.0	22.8	9.6	16.3	25.5

CCL2, MCP-1 (pg/ml)														
Healthy volunteer #		1	2	3	4	5	6	7	8	Median	Mean	SD	Q1 IQR	Q3 IQR
Hours post	0	533.0	489.0	213.0	413.0	324.0	306.0	228.0	456.0					
endotoxin challenge	0	363.0	369.0	236.0	339.0	260.0	281.0	187.0	248.0	315.0	327.8	103.2	245.0	380.0
	1	488.0	808.0	672.0	1016.0	663.0	707.0	428.0	575.0	667.5	669.6	185.5	553.3	732.3
	2	4544.0	4282.0	5745.0	4018.0	3049.0	2999.0	1342.0	5937.0	4150.0	3989.5	1518.6	3036.5	4844.3
	3	5065.0	4805.0	5973.0	4530.0	2548.0	4838.0	1351.0	8346.0	4821.5	4682.0	2102.7	4034.5	5292.0
	4	4430.0	4870.0	5961.0	4099.0	4008.0	3131.0	1773.0	7933.0	4264.5	4525.6	1842.8	3788.8	5142.8
	6	1969.0	3667.0	5089.0	4208.0	4394.0	3662.0	1364.0	4275.0	3937.5	3578.5	1272.6	3238.8	4304.8
	8	779.0	3189.0	1311.0	1566.0	983.0	1276.0	553.0	1815.0	1293.5	1434.0	818.6	932.0	1628.3
	336	550.0	365.0	263.0	375.0	336.0	277.0	175.0	357.0	346.5	337.3	109.1	273.5	367.5

IL-1-RA (pg/ml)														
Healthy volunteer #		1	2	3	4	5	6	7	8	Median	Mean	SD	Q1 IQR	Q3 IQR
Hours post	0	29.0	79.0	118.0	53.0	81.0	50.0	75.0	62.0					
endotoxin challenge	0	11.0	29.0	55.0	36.0	74.0	30.0	69.0	6.0	54.0	53.6	29.3	29.8	74.3
	1	31.0	75.0	118.0	96.0	117.0	71.0	77.0	45.0	76.0	78.8	31.2	64.5	101.3
	2	950.0	2532.0	3438.0	5821.0	1547.0	3141.0	1546.0	2270.0	2401.0	2655.6	1530.7	1546.8	3215.3
	3	*10000.0*	*10000.0*	*10000.0*	*10000.0*	9793.0	*10000.0*	9872.0	9109.0	10000.0	9846.8	308.3	9852.3	10000.0
	4	*10000.0*	*10000.0*	9331.0	*10000.0*	*10000.0*	*10000.0*	*10000.0*	6785.0	10000.0	9514.5	1127.5	9832.8	10000.0
	6	4595.0	5827.0	5001.0	7589.0	4300.0	6525.0	3229.0	2685.0	4798.0	4968.9	1641.2	4032.3	6001.5
	8	2199.0	3647.0	3499.0	6028.0	1846.0	2875.0	1799.0	1930.0	2537.0	2977.9	1434.7	1909.0	3536.0
	336	16.0	25.0	34.0	40.0	79.0	65.0	71.0	16.0	37.0	43.3	25.2	22.8	66.5

IL-10 (pg/ml)														
Healthy volunteer #		1	2	3	4	5	6	7	8	Median	Mean	SD	Q1 IQR	Q3 IQR
Hours post	0	4.0	12.0	35.0	10.0	3.0	10.0	3.0	10.0					
endotoxin challenge	0	3.0	5.0	10.0	4.0	3.0	7.0	3.0	3.0	4.5	7.8	8.0	3.0	10.0
	1	59.0	177.0	136.0	153.0	165.0	146.0	33.0	105.0	141.0	121.8	51.8	93.5	156.0
	2	458.0	407.0	485.0	660.0	395.0	802.0	158.0	572.0	471.5	492.1	192.8	404.0	594.0
	3	684.0	623.0	959.0	597.0	340.0	1134.0	216.0	1162.0	653.5	714.4	348.0	532.8	1002.8
	4	175.0	170.0	379.0	130.0	94.0	256.0	71.0	305.0	172.5	197.5	107.2	121.0	268.3
	6	55.0	92.0	138.0	69.0	74.0	85.0	13.0	37.0	71.5	70.4	37.6	50.5	86.8
	8	29.0	43.0	53.0	54.0	26.0	57.0	10.0	19.0	36.0	36.4	17.8	24.3	53.3
	336	3.0	5.0	6.0	6.0	3.0	11.0	3.0	3.0	4.0	5.0	2.8	3.0	6.0

10000.0 Upper limit value

Uninfected mechanically ventilated ICU patients
Characteristics and cell counts in blood at ICU admission and ICU discharge

		Age	Gender	Reason of admission	ICU stay	Mechanical ventilation	APACHE II score	SOFA score	CRP	PCT	Lactate	HAI	Days till infection	VAP	Non-VAP HAI	Pathogen	Survivor	Days till death
	Patient code	Years	M/F		Days	Yes/No			mg/mL	ng/mL	mmol/L	Yes/No		Yes/No	Yes/No (origin)		Yes/No	
1	1	87	F	Non septic shock	6	Yes	27	15	125	1.78	0.94	Yes	2	Yes	No	Citrobacter freundii	Yes	
2	2	66	M	Non septic shock	21	Yes	24	21	323	51	3	Yes	1	Yes	No	Citrobacter freundii	Yes	
3	3	71	M	Neurological failure	17	Yes	22	10	249	NA	1.06	Yes	2	No	Yes (unknown)	Gram (-) bacillus, enterobacteria	Yes	
4	4	47	F	Neurological failure	20	Yes	19	10	168	NA	0.7	Yes	0	Yes	No	Haemophilus influenzae	Yes	
5	6	68	M	Non septic shock	9	Yes	23	10	69	15.7	3.4	Yes	0	No	Yes (primary bacteremia)	Enterococcus faecalis	Yes	
6	7	71	M	Neurological failure	6	Yes	22	15	NA	NA	NA	No		No	No		No	6
7	8	63	M	Acute respiratory failure	7	Yes	25	9	23	50.3	2.6	Yes	0	Yes	No	Aeromonas spp	Yes	
8	9	48	F	Neurological failure	8	Yes	17	14	76	NA	0.6	Yes	3	Yes	No	Streptococcus aureus	Yes	
9	10	51	M	Neurological failure	6	Yes	13	14	NA	NA	NA	No		No	No		Yes	
10	11	65	F	Neurological failure	11	Yes	16	8	185	0.2	1.3	Yes	1	Yes	No	Klebsiella pneumoniae	Yes	
11	13	52	M	Non septic shock	19	Yes	16	15	322	3.93	1.2	Yes	1	No	Yes (primary bacteremia)	Enterobacter aerogenes	Yes	
12	15	68	F	Neurological failure	8	Yes	23	11	NA	NA	NA	No		No	No		Yes	
13	16	51	F	Neurological failure	11	Yes	16	11	NA	0.06	0.6	Yes	2	No	Yes (unknown)	Unknown	Yes	
14	18	81	F	Non septic shock	14	Yes	21	10	338	3.6	0.8	Yes	3	No	Yes (unknown)	S. epidermidis & C. albicans	Yes	
15	19	63	F	Neurological failure	2	Yes	21	6	NA	NA	NA	No		No	No		Yes	
16	20	32	M	Multiorgan failure	4	Yes	19	17	NA	NA	NA	No		No	No		No	4
17	21	85	F	Neurological failure	6	Yes	20	14	NA	NA	NA	No		No	No		Yes	
18	25	67	M	Non septic shock	19	Yes	23	15	122	0.86	1.8	Yes	0	No	Yes (unknown)	Haemophilus influenzae	Yes	
19	26	84	M	Non septic shock	6	Yes	12	13	162	NA	1.09	Yes	2	Yes	No	Serratia marcescens	Yes	
20	27	65	M	Other	11	Yes	20	14	155	NA	1.7	Yes	0	Yes	No	Unknown	Yes	
21	28	62	M	Non septic shock	22	Yes	11	10	171	0.42	1.1	Yes	8	Yes	No	Haemophilus influenzae	Yes	
22	29	47	M	Neurological failure	5	Yes	18	14	211	0.19	0.9	Yes	0	No	Yes (unknown)	Haemophilus influenzae	Yes	
23	30	69	M	Non septic shock	8	Yes	22	13	110	1.73	3.6	Yes	0	Yes	No	Haemophilus influenzae	Yes	
24	32	66	F	Neurological failure	13	Yes	23	7	103	NA	0.8	Yes	4	No	Yes (unknown)	Proteus mirabilis	No	13
25	33	60	M	Neurological failure	8	Yes	17	8	86	NA	0.6	Yes	6	Yes	No	Haemophilus in	No	8
26	34	65	M	Non septic shock	6	Yes	25	17	121	NA	2.4	Yes	1	No	Yes (urinary)	Enterococcus faecalis	Yes	
27	36	36	F	Acute respiratory failure	4	Yes	18	16	123	NA	1.1	Yes	0	Yes	No	Haemophilus influenzae	Yes	
28	37	61	F	Neurological failure	16	Yes	18	10	281	NA	1.1	Yes	0	Yes	No	Klebsiella aerogenes	Yes	
29	38	52	F	Non septic shock	4	Yes	15	11	NA	NA	0.8	Yes	0	No	Yes (unknown)	Unknown	Yes	
30	39	50	F	Neurological failure	4	Yes	14	5	NA	NA	NA	No		No	No		Yes	
31	42	66	M	Non septic shock	9	Yes	7	6	302	NA	1.1	Yes	5	Yes	No	Haemophilus influenzae	Yes	
32	43	52	M	Other	20	Yes	18	12	358	1.35	0.9	Yes	0	Yes	No	Klebsiella pneumoniae	Yes	
33	44	78	F	Non septic shock	8	Yes	26	15	NA	NA	NA	No		No	No		Yes	
	Median	65.0			8.0		19.0	12.0	162.0	1.7	1.1		1.0					7.0
	Mean	62.2			10.2		19.1	12.0	181.0	9.5	1.4		1.6					7.6
	SD	13.2			5.9		4.6	3.7	97.6	18.5	0.9		2.2					3.5
	Q1 IQR	52.0			6.0		16.0	10.0	115.5	0.4	0.8		0.0					5.5
	Q3 IQR	68.0			14.0		23.0	15.0	265.0	3.9	1.7		2.0					9.3

	At ICU admission	Leukocytes	Neutrophils	Neutrophils	Lin+ cells & DCs	Lin+ cells & DCs	PMN-MDSCs	PMN-MDSCs	Classical monocytes	Classical monocytes	Intermediate & NC monocytes	Intermediate & NC monocytes	M-MDSCs	M-MDSCs	CD15^{low} M-MDSCs	CD15^{low} M-MDSCs	CD15^{int} M-MDSCs	CD15^{int} M-MDSCs
	Patient code	Million cells/mL	Million cells/mL	% of leukocytes	Million cells/mL	% of leukocytes	Million cells/mL	% of leukocytes	Million cells/mL	% of leukocytes	Million cells/mL	% of leukocytes	Million cells/mL	% of leukocytes	Million cells/mL	% of leukocytes	Million cells/mL	% of leukocytes
1	1	x	x	x	x	x	x	x	x	x	x	x	x	x	x	x	x	x
2	2	9.67	8.80	91.03	0.25	2.58	0.05	0.54	0.12	1.25	0.07	0.69	0.37	3.79	0.32	3.32	0.05	0.47
3	3	6.77	5.49	81.10	0.32	4.71	0.23	3.43	0.34	4.99	0.03	0.38	0.32	4.78	0.09	1.26	0.24	3.52
4	4	5.59	4.20	75.17	0.76	13.56	0.18	3.25	0.28	4.95	0.02	0.33	0.03	0.56	0.01	0.12	0.02	0.44
5	6	16.50	15.55	94.22	0.51	3.07	0.03	0.17	0.29	1.77	0.01	0.03	0.12	0.70	0.09	0.57	0.02	0.13
6	7	4.86	3.20	65.93	0.51	10.59	0.50	10.23	0.34	7.09	0.10	2.07	0.19	3.83	0.07	1.52	0.11	2.31
7	8	11.36	7.24	63.73	0.31	2.76	3.07	27.06	0.51	4.52	0.03	0.30	0.17	1.47	0.03	0.24	0.14	1.23
8	9	1.39	0.86	61.75	0.31	22.55	0.03	2.17	0.14	10.00	0.01	0.65	0.03	2.47	0.01	0.52	0.03	1.95
9	10	5.31	0.60	11.25	1.12	21.11	3.30	62.18	0.18	3.36	0.04	0.70	0.05	0.87	0.00	0.01	0.05	0.86
10	11	8.16	6.34	77.67	1.05	12.92	0.01	0.15	0.59	7.28	0.04	0.44	0.05	0.61	0.02	0.30	0.03	0.31
11	13	12.19	11.09	91.00	0.47	3.84	0.04	0.30	0.39	3.20	0.04	0.36	0.14	1.13	0.11	0.93	0.02	0.20
12	15	7.37	6.41	86.91	0.46	6.18	0.04	0.54	0.11	1.55	0.02	0.32	0.27	3.69	0.22	2.97	0.05	0.71
13	16	5.69	4.20	73.76	0.88	15.53	0.01	0.20	0.27	4.68	0.02	0.31	0.08	1.47	0.06	1.00	0.03	0.47
14	18	11.14	9.47	84.99	0.73	6.59	0.06	0.56	0.39	3.49	0.17	1.48	0.09	0.78	0.00	0.04	0.08	0.74
15	19	7.12	4.87	68.41	1.45	20.29	0.02	0.30	0.68	9.58	0.07	0.92	0.03	0.42	0.01	0.07	0.03	0.35
16	20	4.22	1.70	40.26	0.70	16.46	1.54	36.45	0.13	3.17	0.02	0.50	0.10	2.43	0.02	0.56	0.08	1.87
17	21	9.43	6.98	74.02	1.64	17.34	0.25	2.62	0.40	4.28	0.03	0.36	0.11	1.13	0.05	0.52	0.06	0.61
18	25	4.20	0.27	6.55	0.34	8.22	2.77	66.08	0.20	4.80	0.06	1.46	0.53	12.57	0.09	2.14	0.44	10.44
19	26	9.08	6.42	70.74	0.24	2.66	0.24	2.67	1.08	11.90	0.02	0.22	1.05	11.58	1.00	11.04	0.05	0.54
20	27	5.65	1.98	35.10	0.37	6.59	2.93	51.77	0.06	1.06	0.11	1.92	0.20	3.46	0.02	0.44	0.17	3.02
21	28	8.82	1.72	19.53	1.66	18.87	4.65	52.75	0.56	6.41	0.03	0.34	0.14	1.62	0.03	0.29	0.12	1.33
22	29	8.68	5.09	58.59	0.99	11.40	1.85	21.26	0.58	6.68	0.06	0.68	0.04	0.41	0.00	0.02	0.03	0.39
23	30	9.52	3.09	32.44	0.38	4.02	5.19	54.47	0.30	3.13	0.10	1.02	0.46	4.80	0.27	2.85	0.19	1.94
24	32	7.58	1.85	24.44	1.70	22.40	3.05	40.20	0.28	3.70	0.06	0.75	0.29	3.88	0.08	1.08	0.21	2.80
25	33	7.19	4.91	68.29	0.64	8.91	0.82	11.37	0.54	7.50	0.04	0.60	0.19	2.65	0.07	0.95	0.12	1.70
26	34	5.03	3.37	66.89	0.98	19.45	0.09	1.73	0.43	8.56	0.07	1.36	0.06	1.23	0.00	0.02	0.06	1.21
27	36	6.22	3.73	60.01	1.00	16.09	1.20	19.37	0.16	2.58	0.03	0.50	0.07	1.21	0.03	0.53	0.04	0.67
28	37	10.53	7.70	73.13	1.61	15.27	0.28	2.69	0.48	4.57	0.05	0.49	0.24	2.23	0.13	1.28	0.10	0.96
29	38	8.83	7.47	84.62	0.48	5.44	0.16	1.81	0.57	6.49	0.02	0.27	0.09	0.97	0.01	0.14	0.07	0.82
30	39	4.97	3.75	75.56	0.80	16.02	0.08	1.71	0.23	4.68	0.06	1.18	0.02	0.39	0.00	0.03	0.02	0.36
31	42	10.11	7.97	78.77	1.01	10.02	0.02	0.16	0.72	7.16	0.15	1.48	0.18	1.75	0.04	0.40	0.14	1.36
32	43	5.01	3.90	77.93	0.55	11.03	0.13	2.68	0.16	3.12	0.05	1.04	0.19	3.83	0.14	2.75	0.05	1.08
33	44	11.94	11.18	93.66	0.06	0.48	0.29	2.46	0.23	1.90	0.06	0.52	0.11	0.89	0.02	0.15	0.09	0.75
	Median	7.48	4.89	71.94	0.67	10.81	0.24	2.68	0.32	4.63	0.04	0.56	0.13	1.54	0.04	0.53	0.06	0.84
	Mean	7.81	5.34	64.83	0.76	11.14	1.01	14.73	0.37	4.97	0.05	0.73	0.19	2.58	0.09	1.17	0.09	1.41
	SD	3.04	3.45	24.25	0.46	6.68	1.49	21.38	0.22	2.68	0.04	0.51	0.20	2.83	0.18	2.03	0.09	1.86
	Q1 IQR	5.52	3.17	59.66	0.38	5.26	0.05	0.56	0.20	3.16	0.03	0.35	0.07	0.89	0.01	0.15	0.03	0.47
	Q3 IQR	9.56	7.30	79.35	1.00	16.18	1.62	22.71	0.52	6.78	0.06	1.02	0.21	3.71	0.09	1.26	0.12	1.74

	At ICU discharge	Leukocytes	Neutrophils	Neutrophils	Lin+ cells & DCs	Lin+ cells & DCs	PMN-MDSCs	PMN-MDSCs	Classical monocytes	Classical monocytes	Intermediate & NC monocytes	Intermediate & NC monocytes	M-MDSCs	M-MDSCs	CD15^{low} M-MDSCs	CD15^{low} M-MDSCs	CD15^{int} M-MDSCs	CD15^{int} M-MDSCs
	Patient code	Million cells/mL	Million cells/mL	% of leukocytes	Million cells/mL	% of leukocytes	Million cells/mL	% of leukocytes	Million cells/mL	% of leukocytes	Million cells/mL	% of leukocytes	Million cells/mL	% of leukocytes	Million cells/mL	% of leukocytes	Million cells/mL	% of leukocytes
1	1	3.44	2.55	74.23	0.41	11.95	0.00	0.11	0.26	7.66	0.05	1.34	0.04	1.30	0.03	0.79	0.02	0.52
2	2	4.35	2.43	56.01	0.61	14.05	0.12	2.71	0.40	9.16	0.03	0.79	0.08	1.90	0.02	0.43	0.06	1.47
3	3	2.97	1.66	56.08	0.84	28.38	0.01	0.26	0.28	9.59	0.01	0.44	0.03	0.97	0.00	0.08	0.03	0.89
4	4	4.85	3.15	64.90	0.65	13.39	0.52	10.65	0.18	3.74	0.03	0.55	0.19	3.94	0.09	1.77	0.11	2.17
5	8	0.56	0.34	60.56	0.10	18.33	0.07	12.79	0.01	2.20	0.00	0.27	0.01	1.81	0.01	1.27	0.00	0.55
6	9	5.78	4.78	82.76	0.43	7.36	0.01	0.21	0.39	6.83	0.01	0.19	0.08	1.33	0.02	0.43	0.05	0.90
7	13	4.06	3.05	75.06	0.76	18.81	0.00	0.06	0.05	1.12	0.13	3.25	0.02	0.61	0.00	0.02	0.02	0.59
8	16	11.25	9.07	80.64	1.23	10.89	0.01	0.05	0.54	4.84	0.01	0.08	0.19	1.69	0.17	1.54	0.02	0.15
9	18	5.84	4.57	78.16	0.61	10.46	0.02	0.27	0.43	7.30	0.06	1.00	0.01	0.23	0.00	0.00	0.01	0.23
10	19	4.19	2.37	56.39	1.39	33.07	0.02	0.39	0.35	8.36	0.03	0.82	0.02	0.55	0.01	0.21	0.01	0.34
11	25	4.65	3.41	73.32	0.51	11.03	0.13	2.86	0.25	5.29	0.06	1.28	0.13	2.80	0.02	0.40	0.11	2.41
12	26	10.37	7.77	74.90	0.56	5.40	0.13	1.22	1.20	11.54	0.02	0.20	0.62	5.95	0.32	3.04	0.30	2.91
13	28	8.30	1.48	17.79	1.36	16.44	4.47	53.83	0.48	5.74	0.03	0.39	0.11	1.37	0.04	0.52	0.07	0.84
14	37	7.43	5.69	76.58	1.09	14.70	0.14	1.93	0.26	3.50	0.02	0.25	0.02	0.21	0.00	0.01	0.01	0.20
15	39	4.05	1.35	33.39	1.26	31.02	1.03	25.34	0.18	4.51	0.09	2.12	0.01	0.35	0.00	0.03	0.01	0.32
16	42	12.30	10.53	85.60	0.67	5.41	0.01	0.10	0.75	6.10	0.11	0.90	0.04	0.31	0.02	0.13	0.02	0.18
17	44	11.41	10.60	92.86	0.07	0.65	0.05	0.45	0.53	4.63	0.01	0.08	0.07	0.60	0.02	0.16	0.05	0.45
	Median	4.85	3.15	74.23	0.65	13.39	0.05	0.45	0.35	5.74	0.03	0.55	0.04	1.30	0.02	0.40	0.02	0.55
	Mean	6.22	4.40	67.01	0.74	14.79	0.40	6.66	0.38	6.01	0.04	0.82	0.10	1.53	0.04	0.64	0.05	0.89
	SD	3.39	3.25	19.10	0.41	9.03	1.08	13.88	0.28	2.74	0.04	0.83	0.15	1.51	0.08	0.83	0.07	0.85
	Q1 IQR	4.06	2.37	56.39	0.51	10.46	0.01	0.21	0.25	4.51	0.01	0.25	0.02	0.55	0.00	0.08	0.01	0.32
	Q3 IQR	8.30	5.69	78.16	1.09	18.33	0.13	2.86	0.48	7.66	0.06	1.00	0.11	1.81	0.03	0.79	0.06	0.90

NA or x: not available

NC: non classical

Uninfected mechanically ventilated ICU patients
Cytokines in blood

	1	2	3	4	5	6	7	8	9	10	11	12	13	14	15	16	17	18	19	20	21	22
**At ICU admission**	IL-1α	IL-1RA	IL-1β	IL-2	IL-4	IL-5	IL-6	IL-7	IL-9	IL-10	IL-12p70	IL-13	IL-15	IL-17A	IL-18	IL-21	IL-22	IL-23	IL-27	IL-31	IFN-α2	IFN-γ
Patient code	pg/mL	pg/mL	pg/mL	pg/mL	pg/mL	pg/mL	pg/mL	pg/mL	pg/mL	pg/mL	pg/mL	pg/mL	pg/mL	pg/mL	pg/mL	pg/mL	pg/mL	pg/mL	pg/mL	pg/mL	pg/mL	pg/mL
1	x	x	x	x	x	x	x	x	x	x	x	x	x	x	x	x	x	x	x	x	x	x
2	0.0	73301.5	0.0	0.0	0.0	0.0	2920.0	0.0	0.0	20.9	0.0	0.0	0.0	0.0	195.1	0.0	0.0	0.0	0.0	0.0	0.0	0.0
3	4.6	7565.1	41.0	32.6	23.4	17.8	648.6	5.8	0.0	9.4	5.1	15.9	107.5	18.0	115.7	64.6	154.3	0.0	213.9	0.0	0.3	21.5
4	0.4	825.4	20.9	26.9	0.0	3.1	0.0	1.2	0.0	4.0	0.0	10.0	16.1	11.4	72.2	0.0	0.0	0.0	86.7	0.0	0.0	5.0
6	3.7	67011.7	65.5	28.2	67.1	0.0	72.8	0.0	0.0	160.1	11.1	50.2	35.2	0.1	1130.6	72.2	120.2	86.3	307.2	126.7	0.0	97.5
7	1.7	1950.9	0.0	0.0	0.0	0.0	69.7	0.0	0.0	1.9	0.0	0.0	0.0	0.0	65.1	2.3	0.0	0.0	0.0	0.0	0.0	0.0
8	0.0	5263.5	22.9	27.4	3.1	9.1	222.2	2.2	0.0	13.9	4.3	11.2	33.9	8.6	73.1	0.0	0.0	0.0	156.8	0.0	0.0	1.3
9	0.0	367.5	0.0	0.0	0.0	0.0	0.0	0.0	0.0	0.0	0.0	0.0	0.0	0.0	67.8	0.0	0.0	0.0	0.0	0.0	0.0	0.0
10	3.3	279.1	0.0	9.7	0.0	0.0	0.0	0.8	0.0	1.2	0.0	4.9	0.0	0.0	23.4	0.0	90.0	0.0	0.0	0.0	0.0	0.0
11	0.5	1326.2	49.8	50.3	27.1	14.2	0.0	2.8	0.0	6.1	5.7	21.1	48.4	14.7	79.4	0.0	0.0	0.0	234.5	0.0	0.3	17.4
13	5.4	13401.9	1433.1	150.3	1033.7	3.6	533.2	7.5	0.0	24.7	235.6	495.4	365.6	93.3	87.0	40.7	301.3	602.2	3033.7	7.5	19.4	744.0
15	0.5	1677.5	93.3	51.3	44.0	68.1	18.8	3.9	0.0	11.0	6.5	20.4	51.1	23.4	160.4	7.5	0.0	0.0	361.0	0.0	0.0	33.1
16	1.0	530.5	18.2	25.5	3.8	4.3	0.0	2.2	0.0	2.6	4.6	10.8	16.7	7.0	65.1	0.0	74.8	0.0	111.1	0.0	0.0	0.0
18	0.0	3647.0	16.9	27.3	6.6	0.0	0.0	1.9	0.0	5.0	4.7	17.1	45.0	7.4	47.8	0.0	0.0	0.0	141.9	0.0	0.0	0.0
19	3.4	1172.3	42.8	51.5	29.4	15.2	0.0	2.6	0.0	6.3	4.6	25.1	58.8	13.2	83.8	153.0	96.5	66.5	250.3	52.6	0.0	14.1
20	17.6	5699.2	53.1	33.6	1059.5	28.8	533.2	1.6	0.0	9.4	780.6	1997.4	180.7	196.8	155.9	458.2	2651.7	0.0	977.1	0.0	9.5	772.2
21	0.9	1074.4	52.0	33.6	18.3	39.9	172.2	1.8	0.0	5.5	4.7	21.7	55.1	15.3	112.5	0.0	14.2	24.0	239.0	65.7	0.0	14.5
25	0.0	2291.1	16.3	27.3	6.3	5.6	159.4	1.5	0.0	4.3	4.3	13.4	24.7	7.2	74.0	0.0	0.0	0.0	100.4	0.0	0.0	4.4
26	3.0	2169.5	2.5	8.0	0.0	0.0	76.7	0.7	0.0	2.4	4.7	5.2	0.0	0.9	34.9	0.0	0.0	0.0	0.0	0.0	0.0	0.0
27	0.4	8123.4	12.1	15.7	0.0	0.0	912.8	1.6	0.0	15.0	4.1	8.0	15.5	4.3	44.7	0.0	0.0	0.0	63.9	0.0	0.0	0.0
28	5.1	594.7	11.2	23.6	0.0	0.0	0.0	1.7	0.0	3.8	6.2	55.7	35.0	8.6	50.9	24.5	104.3	0.0	334.9	0.0	0.0	0.0
29	0.6	1902.7	49.8	47.5	31.9	21.7	56.2	2.2	0.0	6.8	5.1	21.7	61.5	16.6	79.4	3.4	0.0	14.3	243.5	13.5	0.4	18.4
30	0.8	4386.8	0.0	14.5	0.0	0.0	59.4	1.1	0.0	7.7	4.2	6.7	11.6	3.9	33.1	0.0	0.0	0.0	44.0	0.0	0.0	0.0
32	0.7	1052.4	3.6	21.2	0.0	0.0	0.0	0.9	0.0	1.3	4.4	9.7	35.2	13.1	31.4	0.0	0.0	0.0	257.0	0.0	0.3	0.0
33	0.3	294.2	3.1	15.1	0.0	0.0	0.0	0.9	0.0	1.0	4.2	8.7	20.1	0.1	22.5	0.0	0.0	3.6	42.4	0.0	0.0	0.0
34	125.8	4453.1	915.6	77.5	567.0	0.0	242.9	19.3	0.0	27.1	192.3	416.3	308.3	44.0	54.4	1358.6	5819.6	46.0	1824.9	17.8	13.3	363.6
36	115.0	37812.7	957.5	239.2	942.2	7.6	1262.7	40.1	96.3	98.2	289.5	355.9	397.7	185.2	94.6	1395.9	5480.7	0.0	2351.8	890.5	16.5	575.1
37	0.0	309.2	0.0	0.0	0.0	0.0	0.0	0.6	0.0	0.4	0.0	0.0	0.0	0.0	14.5	0.0	0.0	0.0	0.0	0.0	0.0	0.0
38	0.0	129.7	0.0	12.9	0.0	0.0	0.0	1.8	0.0	0.6	0.0	9.2	11.9	0.0	25.2	0.0	0.0	0.0	10.1	0.0	0.0	0.0
39	0.0	402.7	5.8	12.1	5.0	0.0	0.0	1.8	0.0	1.0	4.0	11.0	21.7	0.7	27.8	0.0	0.0	0.0	79.8	0.0	0.0	0.0
42	0.0	766.4	0.0	0.0	0.0	0.0	75.5	0.5	0.0	3.0	0.0	0.0	0.0	0.0	61.1	0.0	0.0	0.0	25.5	0.0	0.0	0.0
43	0.6	2406.7	43.9	31.5	6.9	48.9	20.1	3.0	0.0	9.2	4.9	12.9	27.6	17.6	127.4	8.5	0.0	0.0	190.6	0.0	0.0	11.1
44	0.0	4249.6	0.0	0.0	0.0	0.0	0.0	1.9	0.0	0.0	0.0	0.0	0.0	0.0	32.2	0.0	0.0	0.0	0.0	0.0	0.0	0.0
Median	0.58	1926.83	16.60	26.17	3.43	0.00	38.14	1.71	0.00	5.24	4.48	11.09	26.15	7.30	66.43	0.00	0.00	0.00	126.49	0.00	0.00	0.00
Mean	8.96	7829.26	119.62	33.95	117.53	8.71	245.29	3.49	2.92	14.21	48.47	110.51	60.94	21.78	103.30	108.76	451.74	25.55	357.83	35.58	1.81	81.61
SD	28.97	17471.45	324.60	46.94	303.89	16.17	563.73	7.42	16.76	31.46	148.86	359.87	102.64	47.12	190.05	337.68	1418.25	105.46	700.27	155.61	5.02	209.55
Q1 IQR	0.00	723.47	0.00	11.51	0.00	0.00	0.00	0.89	0.00	1.77	0.00	6.31	8.69	0.11	34.46	0.00	0.00	0.00	21.68	0.00	0.00	0.00
Q3 IQR	3.34	4655.68	49.76	33.61	27.68	10.35	184.71	2.29	0.00	9.82	5.22	21.72	52.12	15.65	88.85	12.51	91.61	0.00	251.94	0.00	0.06	17.66

**At ICU discharge**	IL-1α	IL-1RA	IL-1β	IL-2	IL-4	IL-5	IL-6	IL-7	IL-9	IL-10	IL-12p70	IL-13	IL-15	IL-17A	IL-18	IL-21	IL-22	IL-23	IL-27	IL-31	IFN-α2	IFN-γ
Patient code	pg/mL	pg/mL	pg/mL	pg/mL	pg/mL	pg/mL	pg/mL	pg/mL	pg/mL	pg/mL	pg/mL	pg/mL	pg/mL	pg/mL	pg/mL	pg/mL	pg/mL	pg/mL	pg/mL	pg/mL	pg/mL	pg/mL
1	0.0	395.7	7.3	11.3	0.0	0.0	0.0	1.7	0.0	4.4	4.3	0.0	13.9	2.3	39.3	0.0	0.0	0.0	0.0	0.0	0.0	0.0
2	0.3	147.9	0.0	0.0	0.0	0.0	0.0	0.0	0.0	3.8	0.0	0.0	0.0	0.0	38.0	0.0	0.0	0.0	0.0	0.0	0.0	0.0
3	10.1	866.1	13.7	21.4	3.4	0.0	0.0	1.0	0.0	2.0	4.7	15.9	100.0	4.3	38.0	43.5	280.7	0.0	100.4	0.0	0.0	1.7
4	0.0	471.1	0.0	6.2	0.0	9.3	36.8	0.0	0.0	2.6	0.0	0.0	0.0	0.0	67.3	0.0	0.0	0.0	0.0	0.0	0.0	0.0
8	0.0	3723.6	0.0	0.0	0.0	0.0	0.0	0.0	0.0	0.5	0.0	0.0	0.0	0.0	49.1	0.0	0.0	0.0	0.0	0.0	0.0	0.0
9	0.6	1702.3	138.9	61.1	47.3	117.3	31.0	5.0	0.0	17.0	5.8	22.3	48.2	39.4	254.1	19.6	0.0	0.0	357.9	0.0	0.3	51.0
13	0.4	2894.7	304.7	45.2	503.9	0.0	0.0	3.9	0.0	5.0	136.1	253.2	227.2	12.8	55.3	0.0	0.0	0.0	1758.8	0.0	9.2	275.5
16	0.9	813.7	16.9	36.9	4.1	0.0	0.0	2.4	0.0	3.2	4.5	14.4	38.9	6.0	50.9	2.1	52.1	0.0	139.4	0.0	0.0	0.9
18	0.9	3128.5	47.6	44.2	27.4	18.3	0.0	4.2	0.0	9.0	5.6	20.9	94.9	17.8	96.4	11.8	0.0	41.0	269.1	16.1	0.8	13.8
19	1.1	1019.1	33.5	45.5	25.1	0.0	0.0	2.2	0.0	5.9	4.5	17.8	50.4	11.3	66.0	29.7	0.0	76.9	218.5	29.9	0.0	8.6
25	0.4	1139.9	46.1	42.2	21.2	20.9	0.0	3.3	0.0	7.3	5.2	18.9	43.7	15.7	101.3	0.0	0.0	0.0	227.7	0.0	0.0	14.9
26	0.4	8955.5	0.0	6.6	0.0	0.0	93.9	2.3	0.0	1.3	0.0	4.3	8.7	0.0	39.3	76.0	0.0	0.0	0.0	0.0	0.0	0.0
28	5.1	594.7	11.2	23.6	0.0	0.0	0.0	1.7	0.0	3.8	6.2	55.7	35.0	8.6	50.9	24.5	104.3	0.0	334.9	0.0	0.0	0.0
37	0.4	1273.6	44.3	44.7	19.4	0.0	0.0	4.0	0.0	5.2	5.2	19.1	60.2	10.8	74.9	0.0	0.0	0.0	227.7	0.0	0.3	7.3
39	0.0	402.7	5.8	12.1	5.0	0.0	0.0	1.8	0.0	1.0	4.0	11.0	21.7	0.7	27.8	0.0	0.0	0.0	79.8	0.0	0.0	0.0
42	1.3	1941.3	22.2	19.9	0.0	0.0	0.0	4.3	0.0	5.3	0.0	14.2	24.7	9.1	102.2	0.0	0.0	0.0	141.9	0.0	0.0	4.3
44	1.2	1030.2	33.2	39.0	10.0	12.1	0.0	3.5	0.0	5.3	4.9	16.8	43.3	11.1	78.5	0.0	0.0	0.0	159.3	0.0	0.0	7.4
Median	0.44	1030.24	16.92	23.63	4.08	0.00	0.00	2.32	0.00	4.38	4.48	15.86	38.93	8.55	55.31	0.00	0.00	0.00	141.91	0.00	0.00	1.66
Mean	1.36	1794.16	42.67	27.05	39.22	10.47	9.51	2.42	0.00	4.85	11.22	28.49	47.69	8.82	72.30	12.18	25.71	6.94	236.20	2.71	0.62	22.66
SD	2.55	2114.34	75.40	19.01	120.50	28.39	24.48	1.60	0.00	3.84	32.25	59.40	54.96	9.82	52.27	21.13	71.20	20.58	410.06	8.03	2.21	66.32
Q1 IQR	0.30	594.72	5.82	11.32	0.00	0.00	0.00	1.66	0.00	2.56	0.00	4.32	13.91	0.74	39.33	0.00	0.00	0.00	0.00	0.00	0.00	0.00
Q3 IQR	1.08	1941.31	44.26	44.19	21.15	9.32	0.00	3.91	0.00	5.32	5.22	19.11	50.42	11.30	78.46	19.56	0.00	0.00	227.68	0.00	0.00	8.58

23	24	25	26	27	28	29	30	31	32	33	34	35	36	37	38	39	40	41	42	43	44	45
**TNF-α**	**CCL2 (MCP-1)**	**CCL3 (MIP-1α)**	**CCL4 (MIP-1β)**	**CCL5 (Rantes)**	**CCL11 (Eotaxin)**	**CXCL1 (Groα/KC)**	**CXCL8 (IL-8)**	**CXCL10 (IP-10)**	**CXCL12 (SDF-1α)**	**TNF-β**	**NGF-β**	**BDNF**	**EGF**	**FGF-2**	**HGF**	**LIF**	**PDGF-BB**	**PlGF-1**	**SCF**	**VEGF-A**	**VEGF-D**	**GM-CSF**
pg/mL	pg/mL	pg/mL	pg/mL	pg/mL	pg/mL	pg/mL	pg/mL	pg/mL	pg/mL	pg/mL	pg/mL	pg/mL	pg/mL	pg/mL	pg/mL	pg/mL	pg/mL	pg/mL	pg/mL	pg/mL	pg/mL	pg/mL
x	x	x	x	x	x	x	x	x	x	x	x	x	x	x	x	x	x	x	x	x	x	x
27.4	419.6	12.4	153.0	141.6	9.4	0.0	230.9	14.3	2148.0	0.0	0.0	53.9	0.0	0.0	15114.5	19.8	20.2	7.7	98.5	3760.4	13.3	0.0
31.2	107.5	184.1	51.9	114.6	24.3	5.0	10.4	41.8	691.7	0.0	5.5	765.4	39.8	16.0	589.0	15.6	1126.0	232.8	11.5	458.3	0.0	206.9
10.3	84.4	0.0	44.1	138.9	14.7	0.0	0.0	13.3	927.4	0.0	0.0	206.6	6.5	2.6	192.2	9.4	100.0	48.9	5.9	123.4	0.0	101.1
32.6	561.0	35.9	241.9	50.0	11.2	8.2	26.5	79.0	2443.8	0.0	16.0	87.8	25.4	23.2	1641.8	4.2	169.3	34.2	6.7	741.3	0.0	209.3
0.0	223.5	6.0	95.2	99.2	38.8	0.0	0.0	17.8	2107.6	0.0	0.0	75.0	0.0	0.0	687.3	5.4	0.0	29.6	14.7	434.8	0.0	0.0
30.0	201.4	0.0	32.9	141.1	15.7	4.7	16.3	13.7	176.6	0.0	3.6	612.8	3.3	7.7	242.6	9.6	553.3	165.4	5.9	137.9	0.0	84.8
0.0	261.8	2.3	109.8	117.9	70.4	0.0	0.0	20.2	2027.4	0.0	0.0	199.9	5.3	0.0	539.5	3.2	15.8	28.7	5.2	128.5	0.0	0.0
7.1	116.3	0.0	28.8	138.2	11.4	0.0	0.0	19.5	396.3	0.0	0.0	45.6	0.0	0.0	157.6	3.8	459.2	80.8	1.7	147.9	0.0	0.0
27.9	29.6	0.0	31.2	123.2	11.1	0.0	0.0	18.0	244.4	0.0	3.4	234.6	18.0	17.7	336.6	12.2	912.2	93.2	8.7	76.9	0.0	265.6
397.3	153.1	15.4	119.2	152.3	44.0	8.7	6.4	70.6	1417.6	0.0	129.1	195.8	142.4	0.0	1973.4	42.1	878.4	150.7	87.6	125.8	79.6	775.7
18.2	43.5	4.2	34.1	103.9	8.9	5.8	0.0	8.7	261.5	0.0	3.7	151.3	12.8	13.1	330.6	24.5	326.6	89.4	10.3	72.9	0.0	303.2
13.5	36.2	0.0	31.2	141.4	10.9	0.0	0.0	20.1	445.4	0.0	3.3	209.4	7.2	0.0	180.0	8.8	577.6	130.5	5.3	201.6	0.0	121.0
23.2	45.8	0.0	19.2	79.9	10.1	0.0	0.0	14.9	87.7	0.0	3.3	137.1	7.4	2.8	1330.4	7.1	1469.9	140.3	8.5	207.8	0.0	110.4
40.1	36.5	15.8	30.1	212.6	19.3	4.9	0.0	12.1	181.5	0.0	0.0	483.2	17.7	27.1	203.7	11.2	607.0	173.9	6.1	301.5	0.0	247.7
1608.8	649.5	12.8	90.0	175.5	26.1	10.4	14.7	46.9	962.7	0.0	73.7	46.5	77.2	7.5	2126.6	87.2	275.5	123.4	23.1	1495.8	195.7	624.7
16.7	27.3	0.7	29.4	290.4	9.2	11.6	10.5	19.1	240.4	0.0	9.3	101.6	8.0	15.5	304.1	14.3	886.1	85.0	11.5	83.9	0.0	192.2
13.8	109.2	0.0	42.4	157.8	26.0	5.0	19.6	24.1	618.9	0.0	3.6	31.2	0.0	0.9	682.1	7.2	62.2	12.2	5.8	199.9	0.0	91.4
6.2	158.8	4.8	60.2	128.5	20.9	0.0	7.6	98.0	1244.9	0.0	5.0	22.1	0.0	0.0	2192.2	5.9	219.5	29.1	13.3	320.2	0.0	0.0
22.2	265.2	0.0	40.9	134.9	16.5	0.0	19.2	26.5	767.5	0.0	0.0	68.6	2.1	0.0	1464.6	8.2	71.9	31.0	8.9	567.4	0.0	23.8
15.9	18.5	0.0	40.9	121.7	15.0	0.0	0.0	17.9	576.6	0.0	0.0	92.0	15.2	34.7	279.6	6.9	54.4	21.6	5.1	139.2	0.0	158.8
32.9	70.1	0.0	24.0	123.9	8.6	0.0	0.0	9.8	159.3	0.0	0.0	289.0	21.0	12.8	397.8	12.2	283.0	121.8	7.9	82.2	0.0	250.0
12.5	89.0	0.0	29.3	109.8	11.6	0.0	2.3	32.1	747.5	0.0	3.2	16.0	1.7	0.0	5085.0	7.5	192.9	14.8	6.2	102.4	0.0	0.0
19.2	70.6	41.4	27.8	113.5	11.9	0.0	0.0	10.0	423.2	0.0	5.2	29.4	8.1	0.0	348.4	14.9	121.7	24.7	2.6	36.7	0.0	43.1
11.6	13.2	0.0	26.1	129.5	7.5	0.0	0.0	6.7	310.2	0.0	0.0	66.3	0.0	1.6	146.0	3.7	437.2	43.1	2.9	15.4	0.0	0.0
160.4	141.1	7.8	214.0	101.3	70.3	11.0	0.0	28.2	1563.8	0.0	44.2	21.8	101.1	15.1	292.9	10.4	172.2	325.9	37.0	1572.1	0.0	123.9
541.3	247.1	22.9	242.3	125.9	31.9	8.4	38.0	29.5	2210.9	0.0	117.1	93.8	137.1	92.6	2185.0	67.5	65.1	523.6	109.3	2041.1	89.7	1240.5
0.0	33.7	0.0	28.2	92.9	11.0	0.0	0.0	9.6	734.2	0.0	0.0	19.8	0.0	0.0	439.5	2.1	190.4	8.1	5.1	34.7	0.0	0.0
0.0	17.2	0.0	19.7	137.4	5.1	0.0	0.0	10.3	253.1	0.0	0.0	69.2	2.4	0.0	134.4	3.6	78.8	20.4	1.4	39.9	0.0	29.1
0.0	17.7	0.0	18.7	156.3	7.2	0.0	0.0	17.3	169.7	0.0	0.0	127.0	3.6	0.0	116.4	4.7	168.5	55.1	1.6	25.1	0.0	81.3
0.0	125.4	0.0	30.2	109.0	10.3	0.0	2.8	37.7	767.5	0.0	3.3	5.9	0.0	0.0	1483.8	3.4	205.1	3.3	6.7	242.1	0.0	0.0
14.2	23.7	19.5	39.9	97.4	13.5	4.5	2.0	15.8	646.8	0.0	5.7	63.8	16.6	0.0	697.7	20.4	510.3	49.7	16.8	174.5	0.0	156.1
0.0	52.8	2.3	28.6	135.9	9.0	0.0	3.8	26.9	720.3	0.0	0.0	9.7	0.0	0.0	3223.9	3.6	19.8	2.3	5.7	154.5	0.0	0.0
16.29	86.69	0.34	33.49	127.18	11.74	0.00	0.00	18.57	669.27	0.00	3.29	81.40	6.84	0.47	489.46	8.49	198.96	49.33	6.67	151.18	0.00	96.26
95.48	137.49	11.78	63.28	131.01	18.89	2.66	12.45	25.72	828.59	0.00	13.37	142.83	20.80	8.82	1382.06	14.21	346.33	89.40	16.79	436.28	11.46	167.78
294.62	154.42	32.68	63.78	46.55	16.34	3.91	40.29	21.31	699.79	0.00	31.87	174.59	37.73	17.66	2698.85	18.40	363.48	108.03	27.34	764.74	38.93	260.48
6.85	35.60	0.00	28.76	109.60	9.92	0.00	0.00	13.59	259.39	0.00	0.00	42.02	0.00	0.00	270.32	4.57	77.04	23.88	5.26	83.43	0.00	0.00
30.30	169.45	12.52	67.62	141.17	21.76	4.95	10.44	28.54	1033.27	0.00	5.30	196.79	17.74	13.58	1523.27	14.41	521.07	125.20	11.97	348.84	0.00	207.50

**TNF-α**	**CCL2 (MCP-1)**	**CCL3 (MIP-1α)**	**CCL4 (MIP-1β)**	**CCL5 (Rantes)**	**CCL11 (Eotaxin)**	**CXCL1 (Groα/KC)**	**CXCL8 (IL-8)**	**CXCL10 (IP-10)**	**CXCL12 (SDF-1α)**	**TNF-β**	**NGF-β**	**BDNF**	**EGF**	**FGF-2**	**HGF**	**LIF**	**PDGF-BB**	**PlGF-1**	**SCF**	**VEGF-A**	**VEGF-D**	**GM-CSF**
pg/mL	pg/mL	pg/mL	pg/mL	pg/mL	pg/mL	pg/mL	pg/mL	pg/mL	pg/mL	pg/mL	pg/mL	pg/mL	pg/mL	pg/mL	pg/mL	pg/mL	pg/mL	pg/mL	pg/mL	pg/mL	pg/mL	pg/mL
0.0	19.9	31.8	32.8	171.1	5.9	0.0	0.0	21.1	578.8	0.0	0.0	101.6	0.0	0.0	233.5	10.8	399.5	42.6	4.0	144.4	0.0	0.0
0.0	89.3	57.3	225.5	146.2	13.1	0.0	6.6	78.6	1990.2	0.0	0.0	55.5	0.0	0.0	997.3	15.3	26.0	5.7	103.2	2042.1	11.5	0.0
9.9	33.0	211.3	46.0	163.7	13.9	0.0	0.0	22.2	335.6	0.0	0.0	219.1	13.5	4.6	217.1	6.1	789.4	218.7	6.0	469.9	0.0	84.8
74.3	685.4	1.5	97.4	50.3	50.5	0.0	0.0	37.9	4162.8	0.0	0.0	382.3	9.4	0.0	1237.5	8.5	140.8	29.3	5.1	233.7	0.0	0.0
0.0	468.9	6.3	130.5	248.7	94.8	0.0	0.0	52.7	1840.2	0.0	0.0	307.4	29.7	0.0	1495.0	4.0	48.0	49.5	26.3	2092.0	0.0	0.0
12.5	36.4	7.1	32.7	111.9	13.6	7.0	0.0	14.9	374.1	0.0	5.4	203.0	28.4	34.2	1413.7	36.0	549.7	137.3	14.4	97.1	0.0	375.6
138.1	48.8	0.0	28.9	89.2	10.0	0.0	0.0	18.4	94.7	0.0	46.6	198.8	63.6	0.0	604.6	8.9	1250.5	131.5	19.5	37.9	0.0	284.1
23.6	15.1	0.0	17.9	126.4	8.2	0.0	0.0	9.2	131.1	0.0	0.0	329.2	11.6	0.0	238.6	7.5	829.5	153.6	3.4	187.2	0.0	158.8
40.8	31.5	0.0	24.6	91.6	10.2	4.2	0.0	8.3	103.2	0.0	4.6	234.4	34.7	16.7	133.7	11.3	1347.0	178.9	7.4	174.8	0.0	253.4
26.4	29.4	4.3	22.4	192.9	17.8	0.0	0.0	10.0	71.3	0.0	0.0	372.7	11.8	9.5	92.8	9.8	563.3	136.1	4.7	216.9	0.0	273.3
19.4	53.4	0.0	35.0	164.6	20.9	10.2	2.3	28.3	335.9	0.0	3.6	424.3	18.8	23.2	292.9	13.7	235.2	103.9	9.3	69.6	0.0	209.3
11.3	9.9	0.0	45.3	183.7	11.7	0.0	2.5	23.1	809.6	0.0	3.6	175.6	12.6	0.3	4280.8	4.8	515.7	92.9	27.3	339.1	0.0	0.0
15.9	18.5	0.0	40.9	121.7	15.0	0.0	0.0	17.9	576.6	0.0	0.0	92.0	15.2	34.7	279.6	6.9	54.4	21.6	5.1	139.2	0.0	158.8
24.1	21.3	0.0	22.2	108.8	6.0	3.5	0.0	7.6	70.5	0.0	0.0	154.6	19.4	5.9	136.4	7.7	96.4	53.0	4.4	35.6	0.0	168.0
0.0	17.7	0.0	18.7	156.3	7.2	0.0	0.0	17.3	169.7	0.0	0.0	127.0	3.6	0.0	116.4	4.7	168.5	55.1	1.6	25.1	0.0	81.3
8.3	36.4	0.0	49.4	188.6	12.4	10.0	2.8	35.6	542.6	0.0	5.2	127.7	22.5	12.8	713.3	8.6	389.9	73.1	11.7	303.6	0.0	0.0
15.0	15.2	0.0	23.4	97.7	7.9	3.5	0.0	9.0	90.2	0.0	0.0	136.6	8.5	0.3	387.9	10.1	122.5	56.1	6.2	102.1	0.0	122.4
14.96	31.53	0.00	32.83	146.20	12.40	0.00	0.00	18.40	335.85	0.00	0.00	198.75	13.50	0.26	292.86	8.61	389.89	73.05	6.22	174.75	0.00	122.43
24.67	95.88	18.79	52.56	141.95	18.76	2.26	0.84	24.24	722.18	0.00	4.06	214.23	17.83	8.35	757.12	10.27	442.72	90.53	15.27	394.71	0.67	127.63
34.49	186.11	51.86	53.43	48.92	22.11	3.62	1.79	18.56	1057.03	0.00	11.18	111.57	15.37	12.02	1023.03	7.28	409.89	60.31	23.97	640.38	2.78	121.18
8.29	18.52	0.00	23.35	108.75	8.23	0.00	0.00	10.04	103.21	0.00	0.00	127.71	9.40	0.00	217.11	6.92	122.46	49.48	4.68	97.06	0.00	0.00
24.11	48.77	6.29	46.03	171.14	15.03	3.53	0.00	28.27	578.77	0.00	3.63	307.40	22.49	12.76	997.26	10.77	563.25	136.14	14.35	303.59	0.00	209.31
